# Neuroimaging and Genetic Markers of Cerebral Small Vessel Disease and Cognitive Outcomes: A Systematic Review and Meta-Analysis (NEUROGEN-SVD Study)

**DOI:** 10.3390/diagnostics15202585

**Published:** 2025-10-13

**Authors:** Chelsea Jin, Roy G. Beran, Sonu M. M. Bhaskar

**Affiliations:** 1 Global Health Neurology Lab, Sydney, NSW 2150, Australia; 2 South West Sydney Clinical Campuses, UNSW Medicine and Health, University of New South Wales (UNSW), Sydney, NSW 2170, Australia; 3 NSW Brain Clot Bank, NSW Health Pathology, Sydney, NSW 2170, Australia; 4 School of Medicine, Western Sydney University, Sydney, NSW 2000, Australia; 5 Griffith Health, School of Medicine and Dentistry, Griffith University, Southport, QLD 4215, Australia; 6 Clinical Sciences Stream, Ingham Institute for Applied Medical Research, Liverpool, NSW 2170, Australia; 7 Department of Neurology & Neurophysiology, Liverpool Hospital and South West Sydney Local Health District, Liverpool, NSW 2150, Australia; 8 Department of Neurology, Division of Cerebrovascular Medicine and Neurology, National Cerebral and Cardiovascular Center (NCVC), Suita 564-8565, Osaka, Japan

**Keywords:** cerebral small vessel disease (CSVD), cognitive impairment, magnetic resonance imaging, APOE ε4, meta-analysis

## Abstract

**Background/Objectives:** Cerebral small vessel disease (CSVD) is a leading cause of cognitive decline and dementia. The comparative prognostic value of MRI-based neuroimaging markers and genetic risk factors such as the APOE ε4 allele for cognitive outcomes remains uncertain. The objectives of this study were to estimate the pooled prevalence of cognitive impairment in CSVD, evaluate the associations of key neuroimaging markers (white matter hyperintensities [WMHs], cerebral microbleeds [CMBs], lacunes) and APOE ε4 with cognitive outcomes, and assess their diagnostic performance. **Methods:** This study included a systematic review and meta-analysis in accordance with PRISMA and MOOSE guidelines, searching five databases (2005–2025). Eligible studies included adults with CSVD and MRI-visible markers reporting cognitive outcomes (mild cognitive impairment [MCI], global cognitive impairment [GCI], all-cause dementia [ACD], vascular dementia [VaD], and Alzheimer’s disease [AD]). Thirty-nine studies comprising 18,425 participants were included. Pooled prevalence and associations were estimated using random-effects models, and diagnostic accuracy was evaluated. Certainty of evidence was assessed using the GRADE framework. **Results:** The pooled prevalence of GCI in CSVD was 57% (95% CI: 51–62%), while MCI prevalence was 46% (95% CI: 42–51%). WMHs were strongly associated with VaD (OR 10.35, 95% CI: 7.32–14.64), lacunes with ACD (OR 3.18, 95% CI: 1.24–8.20), and CMBs with AD (OR 1.52, 95% CI: 1.04–2.24). APOE ε4 carriage increased the risk of GCI (OR 1.80, 95% CI: 1.41–2.29). Across markers, diagnostic sensitivity was low, specificity was moderate-to-high, and AUROC values were modest. GRADE certainty ranged from low to moderate, with the highest confidence for WMHs and VaD. **Conclusions:** CSVD-related MRI markers and APOE ε4 are significantly associated with both early and late cognitive outcomes, supporting the integrated vascular–neurodegenerative continuum. The limited diagnostic sensitivity and variable certainty of evidence highlight the need for harmonized definitions, lesion quantification, and multimodal imaging–genetic approaches to improve early detection and risk stratification of CSVD-related cognitive impairment.

## 1. Introduction

Cerebral small vessel disease (CSVD) encompasses a spectrum of microvascular brain pathologies that play a major role in the development of cognitive impairment and dementia [1,2]. Dementia ranks as the seventh leading cause of mortality globally [3] and is projected to increase in prevalence by 66% by 2050 [4]. CSVD is estimated to contribute to nearly 50% of all dementia cases [5], highlighting the urgency for more accurate diagnostic and predictive tools.

Magnetic resonance imaging (MRI) plays a crucial role in detecting the hallmark features of CSVD [6], including white matter hyperintensities (WMHs) [7], cerebral microbleeds (CMBs) [6,8], and lacunes [9]. The relative associations of these neuroimaging markers with different subtypes and stages of cognitive impairment remain inadequately characterized.

In addition to imaging, genetic risk factors, particularly the apolipoprotein E ε4 (APOE ε4) allele [10], have emerged as important contributors to cognitive decline in CSVD populations [11]. Yet, the combined utility of neuroimaging and genetic profiling in understanding and predicting CSVD-related cognitive outcomes is not well established [12]. Moreover, the mechanistic interplay between vascular injury and neurodegenerative processes, potentially mediated by APOE ε4 [13,14], warrants further investigation [15].

The NEUROGEN-SVD (NEUROimaging and GENetic determinants in Small Vessel Disease–related dementia) study was designed to address key gaps in understanding the relationship between CSVD and cognitive decline through a comprehensive meta-analysis and systematic review. Its objectives are threefold: first, to estimate the pooled prevalence of cognitive impairment among individuals with CSVD; second, to evaluate associations between MRI-based CSVD markers and cognitive outcomes; and third, to assess the impact of genetic risk factors, particularly the APOE ε4 allele, on cognitive impairment in CSVD populations.

## 2. Materials and Methods

### 2.1. Literature Search and Study Selection

**Figure 1:**
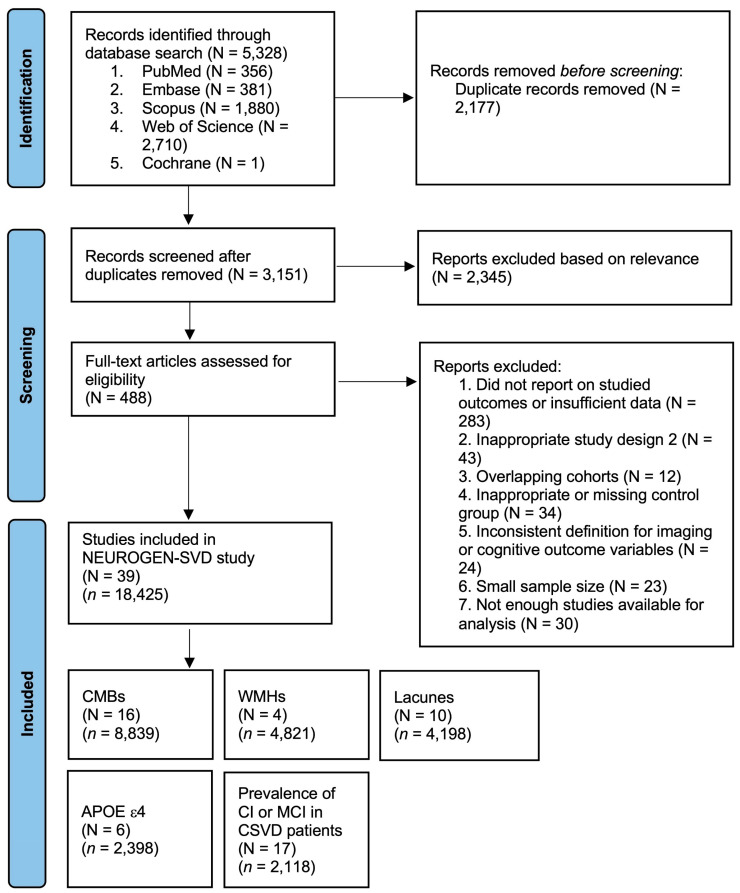
PRISMA flow diagram of study selection process for the NEUROGEN-SVD meta-analysis. The above illustration depicts the study selection flow according to the PRISMA guidelines, leading to the inclusion of studies in the meta-analysis. Abbreviations: NEUROGEN-SVD: NEUROimaging and GENetic determinants in Small Vessel Disease–related dementia; APOE: apolipoprotein E; GCI: global cognitive impairment; CMBs: cerebral microbleeds; CSVD: cerebral small vessel disease; MCI: mild cognitive impairment; WMHs: white matter hyperintensities.

The NEUROGEN-SVD study was conducted in accordance with PRISMA 2020 (Supplemental Table S1) and the Meta-analysis Of Observational Studies in Epidemiology (MOOSE) (Supplemental Table S2) guidelines. A comprehensive search strategy was employed across five databases, namely, PubMed, Embase, Cochrane Library, Scopus, and Web of Science, for studies published between January 2005 and March 2025. The search terms combined keywords and MeSH terms such as “cerebral small vessel disease,” “CSVD,” “MRI,” “APOE ε4,” “vascular cognitive impairment,” and “dementia.” The full search strategy is detailed in the Online Supplemental Information. Study screening and selection were guided by the PRISMA flowchart (Figure 1).

### 2.2. Inclusion and Exclusion Criteria

Inclusion criteria encompassed (a) adult human subjects (≥18 years); (b) the presence of CSVD as defined by STRIVE criteria; (c) cognitive outcomes defined by clear diagnostic criteria; (d) comparative data between cognitively impaired and unimpaired individuals with respect to neuroimaging markers and/or APOE ε4 genotype; and (e) sample size ≥20. Exclusion criteria included non-English publications (unless translated); pediatric/animal studies; case reports; reviews; lack of full text; and studies without extractable data.

### 2.3. Definitions of CSVD Imaging Markers, Genetic Risk Factors, and CSVD

STRIVE [16] neuroimaging standards were used to define the selected imaging markers for CSVD. The markers with sufficient studies meeting eligibility criteria included in this meta-analysis were white matter hyperintensities (WMHs): the presence of moderate-severe (score of 2–3 on the Fazekas [17] scale); cerebral microbleeds (CMBs): the presence of any CMBs; lacunes: the presence of any lacunes; and genetic risk factor: APOE ε4 carrier status. The criteria used to diagnose CSVD varied across included studies and are detailed in Table 1, Table 2 and Table 3.

### 2.4. Definitions of Cognitive Outcomes

The primary outcome was global cognitive impairment (GCI) as defined by each study. Secondary outcomes included mild cognitive impairment (MCI), all-cause dementia (ACD), vascular dementia (VaD), and Alzheimer’s disease (AD). Imaging markers were WMH, lacunes, and CMBs; the genetic marker was APOE ε4 carrier status. Where reported, WMH severity (Fazekas) was captured for dose–response analyses. The diagnostic criteria varied and are detailed in Table 1, Table 2 and Table 3.

### 2.5. Data Extraction

The titles and abstracts of all articles were initially reviewed using Endnote (Clarivate Analytics, London, UK) to exclude articles that did not meet the eligibility criteria. The remaining articles were comprehensively examined to determine their suitability for inclusion in the meta-analysis, in accordance with the defined eligibility criteria. Data extraction was conducted using a dedicated data extraction sheet, recording the following information from each study:

1. Baseline study demographics: author, country, publication year, cohort size, and study design.
2. Patient demographics: age and sex.
3. CSVD neuroimaging marker: WMHs, CMBs, and lacunes.
4. Genetic risk factor: APOE ε4 allele carrier status.
5. CSVD neuroimaging marker and genetic risk factor characteristics: imaging marker score, CSVD diagnostic criteria, and MRI sequence.
6. Cognitive outcome: MCI, ACD, VaD, AD, and GCI.
7. Cognitive outcome characteristics: cognitive diagnostic criteria.

### 2.6. Methodological Quality Assessment of Included Studies

The methodological quality of included studies was assessed using the modified Jadad analysis (MJA) [18], which was completed independently by the primary researcher (Supplemental Table S1). The risk of bias due to funding was also evaluated by assessing the declaration of funding sources and conflicts of interest for each study (Supplemental Table S2).

**Table 1:** Characteristics of prevalence studies included in the NEUROGEN-SVD meta-analysis.

Study ID	Author	Year	Country	Study Design	CSVD Criteria	Cognitive Outcome	GCI Criteria	MCI Criteria	Age (Mean +/− SD)	Female (*n*)	Number of Patients with Cognitive Outcome	Cohort Size	Prevalence
4	Dobrynina et al. [19] (a)	2024	Russia	Cross-sectional	STRIVE	GCI	MOCA	-	59.9 (7.6)	-	111	166	66.9%
4	Dobrynina et al. [19] (b)	2024	Russia	Cross-sectional	STRIVE	MCI	-	DSM-IV	59.9 (7.6)	73	71	166	42.8%
6	Ferro et al. [20]	2017	Netherlands	Cross-sectional	WMHs Fazekas score 2–3, or lacunar infarcts, non-lacunar infarcts, CMBs, intracranial hemorrhage, or WMHs Fazekas 1 and ≥2 vascular risk factors	MCI	-	AHA/ASA VCID	-	-	61	131	46.6%
7	Han et al. [21]	2024	China	Prospective	STRIVE	MCI	-	AHA/ASA VCID	66.2 (6.7)	-	36	69	52.2%
12	Ke et al. [22]	2022	China	Cross-sectional	WMHs Fazekas score 2–3 and/or lacunar infarcts, with or without PVS, CMBs, brain atrophy	GCI	AHA/ASA VCID		-	54	81	137	59.1%
14	Lee et al. [23]	2017	Korea	Prospective	Moderate-severe periventricular WMHs, severe deep WMHs	MCI	Study-specific protocol	Study-specific protocol	74.0 (6.9)	45	33	72	45.8%
17	Liao et al. [24]	2024	China	Cross-sectional	2 or more of WMHs Fazekas score 2–3, lacunes, moderate–severe PVS, CMBs	GCI	MMSE	-	65.9 (10.9)	29	39	94	41.5%
18	Liu et al. [25]	2021	China	Cross-sectional	STRIVE	GCI	Study-specific protocol	-	69.0 (7.8)	92	112	199	56.3%
25	Song et al. [26]	2022	China	Cross-sectional	STRIVE	GCI	NINDS-CSN	-	61.1 (5.0)	45	79	156	50.6%
26	Sun et al. [27]	2022	China	Cross-sectional	WMHs Fazekas score 2–3 and/or lacunar infarct with or without PVS, CMBs, brain atrophy	GCI	MMSE, MOCA	-	-	108	135	242	55.8%
27	Tang et al. [28]	2022	China	Cross-sectional prospective	WMHs Fazekas score 2–3 and at least one of CMBs, lacunes, PVS	GCI	MOCA	-	65.5 (7.7)	48	83	133	62.4%
31	Wang et al. [29]	2023	China	Cross-sectional	WMHs Fazekas score 2–3 OR Fazekas score 1 and vascular risk factors	GCI	MMSE	-	-	14	31	51	60.8%
32	Wei et al. [30]	2019	China	Cross-sectional	WMHs	GCI	MOCA, CDR	-	62.7 (9.1)	53	78	113	69.0%
34	Xing et al. [31]	2021	China	Cross-sectional	WMHs Fazekas score 2–3	MCI	-	DSM-IV	66.4 (6.9)	37	44	77	57.1%
35	Xu et al. [32]	2024	China	Cross-sectional	STRIVE	MCI	-	Study-specific protocol	61.7 (9.2)	76	87	185	47.0%
37	Zhu et al. [33]	2024	China	Cross-sectional	WMHs, lacunes, CMBs, PVS	GCI	MOCA		67.0 (7.0)	71	100	227	44.1%
38	Zhu et al. [34]	2021	China	Cross-sectional	WMHs Fazekas score 3–6 (sum of PVWMHs and DWMHs)	MCI	-	Study-specific protocol	65.5 (6.2)	24	23	66	34.8%

Abbreviations: AHA/ASA VCID: American Heart Association/American Stroke Association Vascular Cognitive Impairment and Dementia; CDR: Clinical Dementia Rating; GCI: global cognitive impairment; CMBs: cerebral microbleeds; CSVD: cerebral small vessel disease; DSM-IV: Diagnostic and Statistical Manual of Mental Disorders, Fourth Edition; DWMHs: deep white matter hyperintensities; MCI: mild cognitive impairment; MMSE: Mini-Mental State Examination; MOCA: Montreal Cognitive Assessment; PVS: perivascular spaces; PVWMHs: periventricular white matter hyperintensities; STRIVE: Standards for Reporting Vascular Changes on Neuroimaging; WMHs: white matter hyperintensities. Note: (a), (b) indicate separate sub-analyses or cohorts from the same study reporting distinct cognitive outcomes.

**Table 2:** Characteristics of studies assessing CSVD neuroimaging markers.

Study ID	Author	Year	Country	Study Design	MRI Sequence	Cohort Size	Cognitive Outcome	MCI Criteria	GCI Criteria	VaD Criteria	AD Criteria	ACD Criteria	Age (Mean +/− SD)	Female (*n*)	Imaging Marker (*n*)	Imaging Marker (%)
CMBs																
2	Chen et al. [35]	2018	China	Prospective	SWI	82	ACD	-	-	-	-	CDR	68.2 (10.1)	20	14	17.1%
3	Ding et al. [36] (a)	2017	Iceland	Prospective	T2 * GRE	2601	ACD	-	-	-	-	DSM-IV	74.6 (4.8)	1532	20	0.8%
3	Ding et al. [36] (b)	2017	Iceland	Prospective	T2 * GRE	2601	VaD	-	-	ADDTC	-	-	74.6 (4.8)	1532	6	0.2%
3	Ding et al. [36] (c)	2017	Iceland	Prospective	T2 * GRE	2601	AD	-	-	-	NINCDS-ADRDA	-	74.6 (4.8)	1532	12	0.5%
5	Fan et al. [37]	2021	China	Cross-sectional	SWI	293	GCI	-	MOCA	-	-	-		174	39	13.3%
8	Hilal et al. [38] (a)	2015	Singapore	Cross-sectional	-	572	GCI	-	Study-specific protocol	-	-	-	70.5 (6.8)	313	152	26.6%
8	Hilal et al. [38] (b)	2015	Singapore	Cross-sectional	-	347	MCI	Study-specific protocol	-	-	-	-	68.6 (5.8)	157	62	17.9%
8	Hilal et al. [38] (c)	2015	Singapore	Cross-sectional	-	204	ACD	-	-	-	-	DSM-IV	68.8 (6.5)	98	12	5.9%
11	Jacob et al. [39] (a)	2023	Netherlands	Prospective	T2 *	498	ACD	-	-	-	-	DSM-IV	65.7 (8.8)	217	22	4.4%
11	Jacob et al. [39] (b)	2023	Netherlands	Prospective	T2 *	424	VaD	-	-	NINDS-AIREN	-	-	64.5 (8.6)	217	14	3.3%
11	Jacob et al. [39] (c)	2023	Netherlands	Prospective	T2 *	428	AD	-	-	-	NIA-AA	-	64.6 (8.7)	217	4	0.9%
15	Li et al. [40]	2021	China	Retrospective	SWI	270	MCI	NIA-AA	-	-	-	-	66.5 (8.7)	118	48	17.8%
17	Liao et al. [24]	2024	China	Cross-sectional	-	94	GCI	-	MMSE	-	-	-	65.9 (10.9)	29	25	26.6%
16	Li et al. [41] (a)	2020	China	Prospective	T2 * GRE	792	MCI	Study-specific protocol	-	-	-	-	72.6 (7.1)	366	124	15.7%
16	Li et al. [41] (b)	2020	China	Prospective	T2 * GRE	792	AD	-	-	-	NINCDS-ADRDA	-	72.6 (7.1)	366	39	4.9%
18	Liu et al. [25]	2021	China	Cross-sectional	-	199	GCI	-	Other	-	-	-	69.0 (7.8)	92	34	17.1%
21	Paradise et al. [42]	2019	Australia	Prospective	SWI	267	ACD	-	-	-	-	DSM-IV/DSM-V	-	-	8	3.0%
23	Romero et al. [43] (a)	2018	US	Prospective	T2 * GRE	1296	ACD	-	-	-	-	DSM-IV	72 (8)	585	17	1.3%
23	Romero et al. [43] (b)	2018	US	Prospective	T2 * GRE	1296	VaD	-	-	NINDS-AIREN	-	-	72 (8)	586	4	0.3%
23	Romero et al. [43] (c)	2018	US	Prospective	T2 * GRE	1296	AD	-	-	-	NINCDS-ADRDA	-	72 (8)	587	13	1.0%
24	Shaikh et al. [44] (a)	2022	US	Cross-sectional	-	94	MCI	NIA-AA	-	-	-	-	69.6 (7.5)	52	7	7.4%
24	Shaikh et al. [44] (b)	2022	US	Cross-sectional	-	89	AD	-	-	-	NIA-AA	-	68.6 (6.9)	52	1	1.1%
28	Uetani et al. [45] (a)	2013	Japan	Cross-sectional	SWI	347	GCI	International Working Group on MCI	-	NINDS-AIREN	NINCDS-ADRDA	-	74.3 (8.8)	217	153	44.1%
28	Uetani et al. [45] (b)	2013	Japan	Cross-sectional	SWI	83	MCI	International Working Group on MCI	-	-	-	-	74.1 (9.5)	55	21	25.3%
28	Uetani et al. [45] (c)	2013	Japan	Cross-sectional	SWI	296	ACD	-	-	NINDS-AIREN	NINCDS-ADRDA	-	-	188	132	44.6%
28	Uetani et al. [45] (d)	2013	Japan	Cross-sectional	SWI	60	VaD	-	-	NINDS-AIREN	-	-	73.3 (10.0)	40	24	40.0%
28	Uetani et al. [45] (e)	2013	Japan	Cross-sectional	SWI	194	AD	-	-	NINDS-AIREN	NINCDS-ADRDA	-	74.3 (9.5)	134	77	39.7%
33	Wrigley et al. [46] (a)	2024	Australia	Retrospective	SWI	219	MCI	Study-specific protocol	-	-	-	-	-	112	37	16.9%
33	Wrigley et al. [46] (b)	2024	Australia	Retrospective	SWI	234	ACD	-	-	-	-	Study-specific protocol	-	115	50	21.4%
35	Xu et al. [32]	2024	China	Retrospective		185	MCI	Study-specific protocol	-	-	-	-	61.7 (9.2)	76	18	9.7%
39	Zonneveld et al. [47] (a)	2014	Netherlands	Cross-sectional	SWI	311	MCI	Petersen’s criteria	-	-	-	-	64.2 (10.2)	125	40	12.9%
39	Zonneveld et al. [47] (b)	2014	Netherlands	Cross-sectional	SWI	509	ACD	-	-	-	-	Other	-	-	107	21.0%
39	Zonneveld et al. [47] (c)	2014	Netherlands	Cross-sectional	SWI	180	VaD	-	-	NINDS-AIREN	-	-	61.5 (10.0)	80	9	5.0%
39	Zonneveld et al. [47] (d)	2014	Netherlands	Cross-sectional	SWI	417	AD	-	-	-	NINCDS-ADRDA		65.2 (10.0)	204	79	18.9%
Moderate–severe WMHs																
4	Dobrynina et al. [19]	2024	Russia	Cross-sectional	-	126	MCI	DSM-V	-	-	-	-	59.9 (7.6)	73	64	50.8%
22	Rennie et al. [48] (a)	2024	Sweden	Cross-sectional	-	2994	MCI	ICD-10	-	-	-	-	70.2 (9.2)	1592	388	13.0%
22	Rennie et al. [48] (b)	2024	Sweden	Cross-sectional	-	2213	AD	-	-	-	ICD-10	-	69.0 (8.6)	1299	188	8.5%
22	Rennie et al. [48] (c)	2024	Sweden	Cross-sectional	-	1653	VaD	-	-	ICD-10	-	-	68.1 (8.4)	942	92	5.6%
28	Uetani et al. [45] (a)	2013	Japan	Cross-sectional	-	83	MCI	International Working Group on MCI	-	-	-	-	74.1 (9.5)	55	20	24.1%
28	Uetani et al. [45] (b)	2013	Japan	Cross-sectional	-	194	AD	-	-	-	NINCDS-ADRDA	-	74.3 (9.5)	134	64	33.0%
28	Uetani et al. [45] (c)	2013	Japan	Cross-sectional	-	60	VaD	-	-	NINDS-AIREN	-	-	73.3 (10.0)	40	24	40.0%
39	Zonneveld et al. [47] (a)	2014	Netherlands	Cross-sectional	-	311	MCI	Petersen’s criteria	-	-	-	-	64.2 (10.2)	125	44	14.1%
39	Zonneveld et al. [47] (b)	2014	Netherlands	Cross-sectional	-	417	AD	-	-	-	NINCDS-ADRDA	-	65.2 (10.0)	204	81	19.4%
39	Zonneveld et al. [47] (c)	2014	Netherlands	Cross-sectional	-	180	VaD	-	-	NINDS-AIREN	-	-	61.5 (10.0)	80	10	5.6%
Lacunes																
5	Fan et al. [37]	2021	China	Retrospective	T1, T2, FLAIR, DWI	293	GCI	-	MOCA	-	-	-	-	174	130	44.4%
8	Hilal et al. [38]	2015	Singapore	Cross-sectional	T2, FLAIR	204	ACD	-	-	-	-	DSM-IV	68.8 (6.5)	98	13	2.3%
8	Hilal et al. [38]	2015	Singapore	Cross-sectional	T2, FLAIR	572	GCI	-	Study-specific protocol	-	-	DSM-IV	70.5 (6.8)	313	99	17.3%
9	Hilal et al. [49]	2021	Singapore	Cross-sectional	T1, T2, FLAIR	253	MCI	Study-specific protocol	-	-	-	-	70.2 (6.1)	129	36	14.2%
11	Jacob et al. [39]	2023	Netherlands	Prospective	T1, FLAIR	498	ACD	-	-	-	-	DSM-V	65.7 (8.8)	217	42	8.4%
15	Li et al. [40]	2021	China	Retrospective	-	270	MCI	NIA-AA	MMSE	-	-	-	66.5 (8.7)	-	118	15.2%
17	Liao et al. [24]	2024	China	Cross-sectional	-	94	GCI	-	MMSE	-	-	-	65.9 (10.9)	-	29	37.2%
18	Liu et al. [25]	2021	China	Cross-sectional	-	199	GCI	-	Study-specific protocol	-	-	-	69.0 (7.8)	-	85	42.7%
28	Uetani et al. [45] (a)	2013	Japan	Cross-sectional	T2WI GRE FLAIR	296	ACD	-	-	NINDS-AIREN	NINCDS-ADRDA	-	-	188	58	19.6%
28	Uetani et al. [45] (b)	2013	Japan	Cross-sectional	T2WI GRE FLAIR	83	MCI	International Working Group on MCI	-	-	-	-	74.1 (9.5)	-	13	15.7%
28	Uetani et al. [45] (c)	2013	Japan	Cross-sectional	T2WI GRE FLAIR	347	GCI	International Working Group on MCI	-	NINDS-AIREN	NINCDS-ADRDA	-	74.3 (8.8)	-	217	20.5%
29	Wang et al. [50]	2022	China	Cross-sectional	-	442	ACD	-	-	-	-	DSM-IV	71.6 (11.3)	-	205	10.9%
30	Wang et al. [51]	2024	China	Cross-sectional	FLAIR	1230	MCI	Petersen’s criteria	-	-	-	-	69.4 (4.3)	-	720	8.0%

Abbreviations: ACD: all-cause dementia; AD: Alzheimer’s Disease; ADDTC: Alzheimer’s Disease Diagnostic and Treatment Centers; CDR: Clinical Dementia Rating; GCI: global cognitive impairment; CMB: cerebral microbleed; CSVD: cerebral small vessel disease; DSM-IV: Diagnostic and Statistical Manual of Mental Disorders, Fourth Edition; DSM-V: Diagnostic and Statistical Manual of Mental Disorders, Fifth Edition; DWI: Diffusion-weighted Imaging; FLAIR: Fluid-Attenuated Inversion Recovery; GRE: Gradient-recalled Echo; ICD-10: International Classification of Diseases, 10th Revision; MCI: mild cognitive impairment; MMSE: Mini-Mental State Examination; MOCA: Montreal Cognitive Assessment; MRI: Magnetic Resonance Imaging; NIA-AA: National Institute on Aging and Alzheimer’s Association; NINCDS-ADRDA: National Institute of Neurological and Communicate Disorders and Stroke/Alzheimer’s Disease and Related Disorders Association; NINDS-AIREN: National Institute of Neurological Disorders and Stroke/Association Internationale pour la Recherche et I’Enseignement en Neurosciences; PVS: perivascular spaces; STRIVE: Standards for Reporting Vascular Changes on Neuroimaging; SWI: Susceptibility-weighted Imaging; T2WI: T2-weighted Imaging; VaD: vascular dementia; WMH: white matter hyperintensities. Notes: The symbol “*” in MRI sequence (e.g., T2* GRE) indicates gradient-echo T2-weighted imaging sequences used for susceptibility detection (commonly employed for visualizing cerebral microbleeds); (a), (b), and (c) indicate separate sub-analyses or cohorts from the same study reporting distinct cognitive outcomes.

**Table 3:** Characteristics of studies assessing APOE ε4 carrier status.

Study ID	Author	Year	Country	Study Design	Cohort Size	Cognitive Outcome	GCI Criteria	Age (Mean +/− SD)	Female (*n*)	APOE ε4 Carrier (*n*)	APOE ε4 Carrier (%)
1	Brickman et al. [52]	2015	USA	Cross-sectional	694	GCI	DSM-IV	80.4 (5.7)	462	14	2.0%
10	Hong et al. [53]	2011	Korea	Cross-sectional	216	GCI	DSM-IV	68.4 (9.3)	161	17	7.9%
13	Kim et al. [54]	2013	Korea	Cross-sectional	364	GCI	DSM-IV, Petersen’s criteria	68.3 (8.5)	212	35	9.6%
19	Nicoll et al. [13]	2010	UK	Prospective	310	GCI	Neuropathological	-	188	56	18.1%
20	Paradela et al. [14]	2023	Brazil	Prospective	648	GCI	CDR	74.7 (12.0)	339	76	11.7%
36	Yu et al. [55]	2023	China	Cross-sectional	166	GCI	CDR	76.5 (7.7)	101	19	11.4%

Abbreviations: APOE: apolipoprotein E; CDR: Clinical Dementia Rating; GCI: global cognitive impairment; DSM-IV: Diagnostic and Statistical Manual of Mental Disorders, Fourth Edition.

### 2.7. Certainty of Evidence Assessment (NEUROGEN-SVD)

As part of the NEUROGEN-SVD study, the certainty of evidence for each cognitive outcome was evaluated using the Grading of Recommendations, Assessment, Development and Evaluations (GRADE) framework. Outcomes were assessed for risk of bias (study design limitations and diagnostic variability), inconsistency (heterogeneity in effect estimates), indirectness (applicability of study populations and outcome definitions), imprecision (width of confidence intervals and sample size), and publication bias (funnel plots, Egger’s, and Deek’s tests). A NEUROGEN-SVD Summary of Findings (SoF) table was constructed, presenting pooled effect estimates, absolute effects, and certainty ratings for the associations between CSVD neuroimaging/genetic markers and cognitive outcomes.

### 2.8. Statistical Analyses

All statistical analyses were conducted using STATA version 13.0 (StataCorp, College Station, TX, USA). Descriptive statistics were used to summarize baseline characteristics and key outcomes across included studies. Where necessary, means and standard deviations (SDs) were imputed from medians and interquartile ranges (IQRs) using the method described by Wan et al. [56].

The pooled prevalence of GCI among patients with CSVD was calculated using the metaprop command, implementing a random-effects model to account for inter-study variability. Exact binomial methods (cimethod(exact) and ftt) were used to derive 95% confidence intervals (CIs) for prevalence estimates. To estimate the associations between CSVD markers (neuroimaging and genetic) and cognitive outcomes, a random-effects meta-analysis using the DerSimonian-Laird (DL) method was applied via the metan package. Exploratory subgroup analyses were performed when at least three or more studies reported comparable data for a given exposure–outcome pair (e.g., imaging marker type and dementia subtype). However, stratified subgroup analyses by diagnostic framework, MRI modality, or cohort setting, as well as meta-regression, were not feasible because most studies did not report compatible covariates. Results were reported as pooled odds ratios (ORs) with corresponding 95% CIs and visualized using forest plots, which also detailed study weights and between-study heterogeneity.

The midas package in STATA was used to evaluate diagnostic performance. Summary receiver operating characteristic (SROC) curves were generated, and pooled estimates of sensitivity, specificity, and area under the ROC curve (AUROC) were reported. Statistical heterogeneity was assessed using the I^2^ statistic (with the following thresholds: <30% = low, 30–50% = moderate, 50–75% = substantial, >75% = high) and Cochran’s Q test. Between-study variance was estimated using Tau^2^. Sensitivity analyses were performed using the metaninf function to assess the influence of individual studies on pooled effect sizes. Funnel plots were generated to visually assess asymmetry, and Egger’s test was applied using the metabias and metafunnel packages to formally test for publication bias. Additional assessments included Deek’s funnel-plot asymmetry test and Fagan’s nomogram (implemented through the midas package) to evaluate potential bias in diagnostic accuracy estimates. All statistical tests were two-tailed, and *p* < 0.05 was considered statistically significant.

## 3. Results

### 3.1. Description of Included Studies

The systematic search across five major databases identified 5328 records. Following the removal of duplicates, 3151 unique records were retained for screening. Abstracts were reviewed for relevance, resulting in the exclusion of 2345 studies that did not address CSVD, cognitive outcomes, or genetic risk factors. Full-text review of the remaining 488 studies led to the exclusion of 449 for the following reasons: absence of relevant outcomes or insufficient data (*n* = 283); inappropriate study design (*n* = 43); overlapping cohorts (*n* = 12); unsuitable control groups (*n* = 34); unclear or inconsistent definitions of imaging or cognitive variables (*n* = 24); small sample size below eligibility thresholds (*n* = 23); and imaging or genetic markers with insufficient numbers of studies for pooled analysis (*n* = 30).

Thirty-nine (39) studies met all inclusion criteria and were included in the final meta-analysis, encompassing 18,425 participants. Where data from the same database were available across multiple reports, priority was given to the largest or most recent cohort to avoid duplication. When data originated from the same database but were reported on distinct subgroups or different imaging/genetic markers, overlapping participants were excluded when calculating the total sample size.

Of the included studies, 16 reported on CMBs, 4 focused on moderate-to-severe WMHs, 10 on lacunes, 6 on APOE ε4 allele carrier status, and 17 provided prevalence estimates of cognitive outcomes in CSVD populations. These studies covered a wide geographic span, including Asian, European, and North American cohorts, enhancing generalizability. Clinical characteristics, diagnostic definitions, and cognitive outcome measures are detailed in Table 1, Table 2 and Table 3.

**Figure 2:**
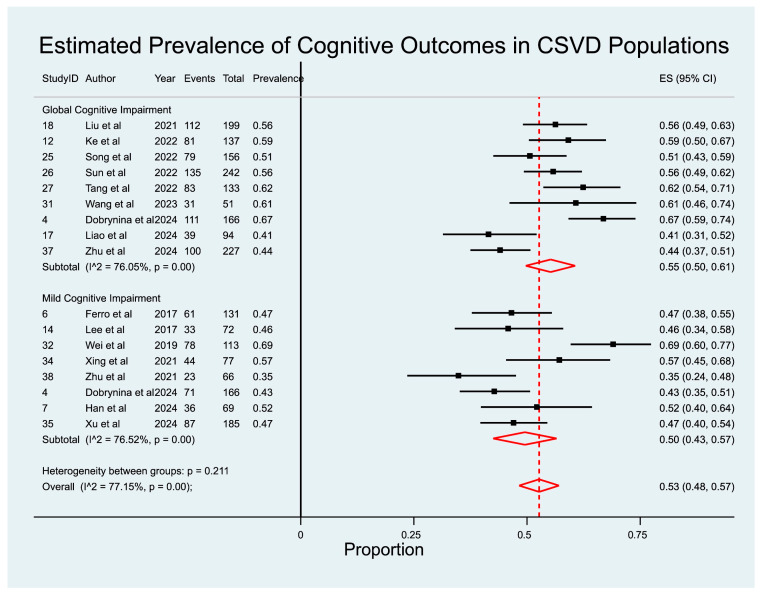
Forest plot of pooled prevalence of cognitive outcomes in CSVD patients. Abbreviations: CSVD: cerebral small vessel disease [19,20,21,22,23,24,25,26,27,28,29,30,31,32,33,34].

**Figure 3:**
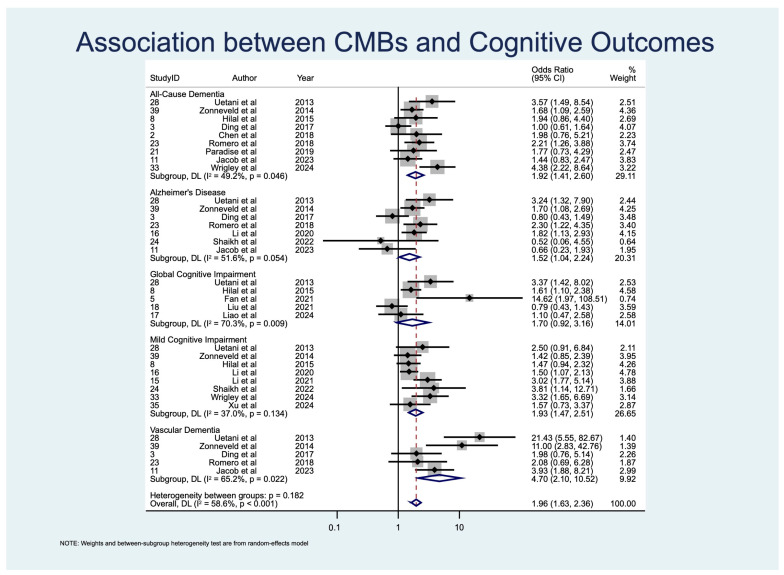
Forest plot of associations between cerebral microbleeds (CMBs) and cognitive outcomes. Abbreviations: CMBs: cerebral microbleeds [24,25,32,35,36,37,38,39,40,41,42,43,44,45,46,47].

**Figure 4:**
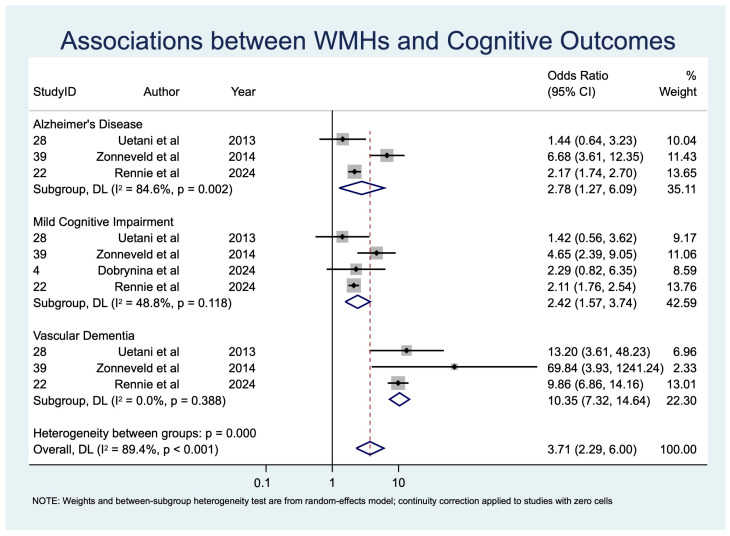
Forest plot of associations between moderate-to-severe white matter hyperintensities (WMHs) and cognitive outcomes. Abbreviations: WMHs: white matter hyperintensities [19,45,47,48].

**Figure 5:**
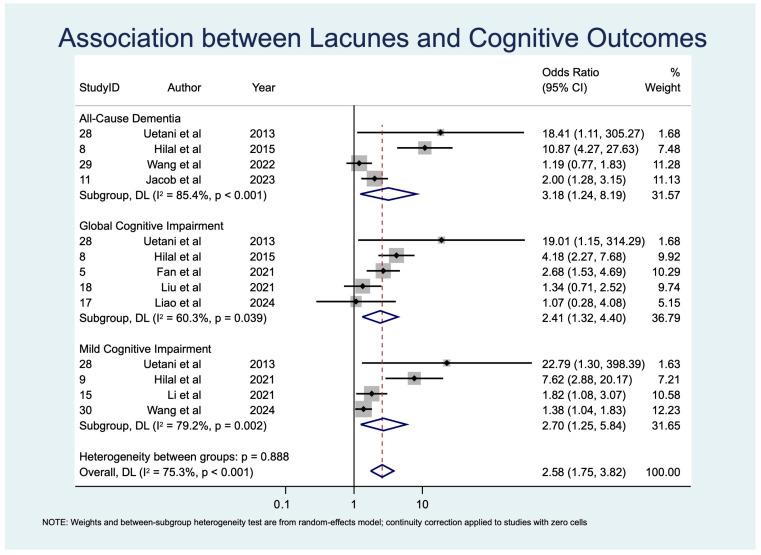
Forest plot of associations between lacunes and cognitive outcomes [24,25,37,38,39,40,45,49,50,51].

**Figure 6:**
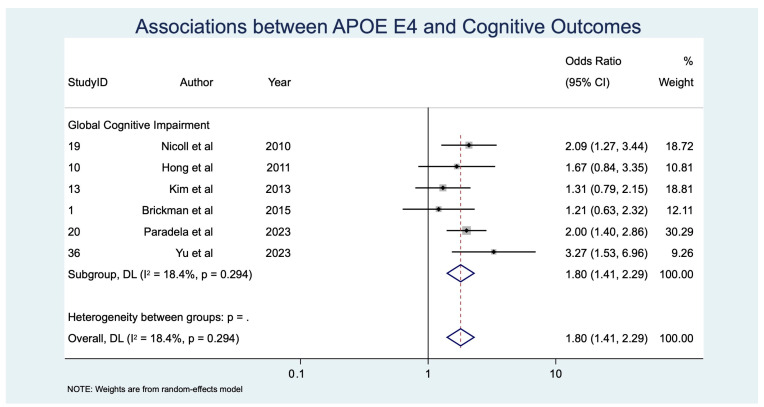
Forest plot of associations between APOE ε4 carrier status and cognitive outcomes [13,14,52,53,54,55]. Abbreviations: APOE: apolipoprotein E.

Pooled prevalence estimates of cognitive impairment outcomes are summarized in Table 4 and displayed as a forest plot in Figure 2. Associations between individual CSVD imaging markers and APOE ε4 status with cognitive outcomes are provided in Table 5 and Table 6, organized by imaging/genetic risk factor (Table 5) and by cognitive outcome (Table 6). Corresponding odds ratios, study weights, and heterogeneity estimates are presented in Figure 3, Figure 4, Figure 5 and Figure 6.

Although not the primary focus of this analysis, the diagnostic performance of imaging and genetic markers was also examined. These results, including pooled sensitivity, specificity, and AUROC estimates, are presented in Supplemental Table S5 and visualized through SROC curves in Supplemental Figures S1 and S2.

The methodological quality of included studies was appraised using a modified Jadad scale (Supplemental Table S3), while potential funding-related bias was assessed in  Supplemental Table S4. Sensitivity analyses were conducted to evaluate the influence of individual studies on pooled results, with findings displayed in Supplemental Figure S3. To assess publication bias, funnel plots, Egger’s regression, and Deek’s funnel-plot asymmetry test were applied, with results presented in Supplemental Tables S6 and S7 and Supplemental Figures S4–S6. Fagan’s nomogram analysis, included for exploratory purposes, is shown in Supplemental Figure S7.

### 3.2. Prevalence of Cognitive Outcomes in CSVD Patients

Sixteen studies [19,20,21,22,23,24,25,26,27,28,29,30,31,32,33,34], comprising a total of 2118 participants, were included in the pooled prevalence analysis of cognitive outcomes in patients with CSVD (Figure 2). The overall meta-analysis demonstrated that cognitive impairment was present in more than half of individuals with CSVD, with a pooled prevalence of 53% (95% CI: 49–58%; z = 33.88; *p* < 0.001) (Table 6; Figure 2). Between-study heterogeneity was substantial (I^2^ = 76.15%, *p* < 0.001), reflecting differences in study populations, diagnostic thresholds, and neuropsychological batteries. Figure 2 provides a stratified visualization of prevalence estimates by cognitive outcome subtype, specifically distinguishing between clinically defined GCI and MCI.

#### 3.2.1. Prevalence of GCI in CSVD Patients

Ten studies [19,22,24,25,26,27,28,29,30,33] (*n* = 1518) specifically assessed the prevalence of GCI among CSVD patients. The pooled prevalence was 57% (95% CI: 51–62%; z = 28.48; *p* < 0.001), indicating that nearly three out of five patients with radiological evidence of CSVD exhibit a measurable global cognitive decline (Table 6; Figure 2). Heterogeneity across these studies was high (I^2^ = 78.65%, *p* < 0.001), suggesting variability in diagnostic methods and cohort characteristics. Some studies drew participants from memory clinics and stroke registries, whereas others used community-based samples, which may partly explain the observed inconsistency. Despite this heterogeneity, the direction of effect was consistent, underscoring GCI as a common and clinically significant outcome of CSVD.

#### 3.2.2. Prevalence of MCI in CSVD Patients

Seven studies [19,20,21,23,31,32,34] (*n* = 766) examined the prevalence of MCI among CSVD patients. The pooled prevalence was 46% (95% CI: 42–51%; z = 31.02; *p* < 0.001), with only moderate heterogeneity (I^2^ = 32.67%, *p* = 0.18) (Table 6; Figure 2). This finding suggests that almost half of CSVD patients exhibit early-stage cognitive decline, often preceding overt dementia. The lower heterogeneity compared to GCI analyses may reflect more consistent use of standardized MCI criteria, such as Petersen’s framework or modified NINDS-CSN definitions, across contributing studies.

#### 3.2.3. Geographical Subgroup Analysis

When stratified by region, notable differences emerged. Asian cohorts (predominantly from China, Korea, and Singapore) reported a pooled prevalence of GCI around 60–65%, often higher than Western cohorts (Europe and North America), where prevalence estimates clustered between 45 and 55%. For MCI, Asian studies reported slightly higher prevalence (48–50%) compared to Western studies (42–45%), though heterogeneity was lower overall. This suggests that cultural and methodological differences may partly explain variability in GCI prevalence, whereas MCI prevalence estimates appear more stable across regions.

### 3.3. Association Between Neuroimaging Markers of CSVD and the APOE ε4 Allele with Cognitive Outcomes

#### 3.3.1. Analysis by Imaging Marker

##### Cerebral Microbleeds (CMBs)

Sixteen studies [24,25,32,35,36,37,38,39,40,41,42,43,44,45,46,47] (*n* = 8612) examined the association between CMBs and cognitive outcomes. The presence of CMBs was significantly associated with MCI (OR 1.93, 95% CI: 1.48–2.51, *p* < 0.001), all-cause dementia (ACD; OR 1.92, 95% CI: 1.41–2.60, *p* < 0.001), vascular dementia (VaD; OR 4.70, 95% CI: 2.10–10.52, *p* < 0.001), and AD (OR 1.52, 95% CI: 1.04–2.24, *p* = 0.033). No statistically significant association was observed with overall GCI (OR 1.70, 95% CI: 0.92–3.16, *p* = 0.091) (Table 5; Figure 3). Heterogeneity ranged from moderate for MCI (I^2^ = 37.0%) and ACD (I^2^ = 49.2%) to substantial for VaD (I^2^ = 65.2%) and GCI (I^2^ = 70.3%), and was highest for AD (I^2^ = 80.8%).

##### White Matter Hyperintensities (WMHs)

Four studies [19,45,47,48] (*n* = 4821) investigated moderate-to-severe WMHs. Strong associations were found with MCI (OR 2.42, 95% CI: 1.57–3.74, *p* < 0.001), VaD (OR 10.35, 95% CI: 7.32–14.64, *p* < 0.001), and AD (OR 2.78, 95% CI: 1.27–6.09, *p* = 0.011) (Table 5; Figure 4). Heterogeneity was low for VaD (I^2^ = 0.0%), moderate for MCI (I^2^ = 48.8%), and high for AD (I^2^ = 84.6%). These findings suggest WMHs are a particularly robust imaging marker for vascular dementia and exert significant influence on both early and late cognitive outcomes.

##### Lacunes

Ten studies [24,25,37,38,39,40,45,49,50,51] (*n* = 4198) assessed lacunes, showing significant associations with MCI (OR 2.70, 95% CI: 1.25–5.84, *p* = 0.011), GCI (OR 2.41, 95% CI: 1.33–4.40, *p* = 0.004), and ACD (OR 3.18, 95% CI: 1.24–8.20, *p* = 0.017) (Table 5; Figure 5). Heterogeneity was substantial for GCI (I^2^ = 60.3%) and high for both MCI (I^2^ = 79.2%) and ACD (I^2^ = 85.4%). This likely reflects differences in imaging protocols, diagnostic thresholds, and classification challenges in distinguishing lacunes from enlarged perivascular spaces.

##### APOE ε4 Allele

Six studies [13,14,52,53,54,55] (*n* = 2398) examined APOE ε4 carrier status. Carriers had significantly higher odds of GCI (OR 1.80, 95% CI: 1.41–2.29, *p* < 0.001) (Table 5; Figure 6). Heterogeneity was substantial (I^2^ = 68.3%, *p* = 0.017), likely reflecting population differences in allele frequency and interactions with vascular risk factors.

#### 3.3.2. Analysis by Cognitive Outcome

*MCI:* Twelve studies [19,32,38,40,41,44,45,46,47,48,49,51] (*n* = 6904) indicated that lacunes (OR 2.70, 95% CI: 1.25–5.84), WMHs (OR 2.42, 95% CI: 1.57–3.74), and CMBs (OR 1.93, 95% CI: 1.48–2.51) were significantly associated with MCI (Table 6).

*GCI*: Eleven studies [13,14,24,25,37,38,45,52,53,54,55] (*n* = 3903) showed that lacunes (OR 2.41, 95% CI: 1.33–4.40) and APOE ε4 allele status (OR 1.80, 95% CI: 1.41–2.29) were the strongest predictors of GCI. No significant association was observed with CMBs (OR 1.70, 95% CI: 0.92–3.16, *p* = 0.091) (Table 6).

*ACD:* Ten studies [35,36,38,39,42,43,45,46,47,50] (*n* = 6429) demonstrated significant associations with lacunes (OR 3.18, 95% CI: 1.24–8.20) and CMBs (OR 1.92, 95% CI: 1.41–2.60) (Table 6).

*VaD*: Six studies [36,39,43,45,47,48] (*n* = 6214) found very strong associations with WMHs (OR 10.35, 95% CI: 7.32–14.64) and CMBs (OR 4.70, 95% CI: 2.10–10.52) (Table 6).

*AD*: Eight studies [36,39,41,43,44,45,47,48] (*n* = 8030) reported significant associations with WMHs (OR 2.78, 95% CI: 1.27–6.09) and CMBs (OR 1.52, 95% CI: 1.04–2.24) (Table 6).

Publication bias was formally assessed using funnel plots, Egger’s regression, and Deek’s test. Visual inspection of funnel plots suggested small-study effects in some analyses, particularly for lacunes and APOE ε4. Egger’s test was significant in a subset of comparisons, while Deek’s test indicated asymmetry in diagnostic accuracy analyses for CMBs and lacunes but not for WMHs or APOE ε4 (Supplemental Tables S6 and S7; Supplemental Figures S4–S6).

These findings suggest that some degree of publication bias may be present, particularly for markers with fewer contributing studies, but overall results remained directionally consistent across sensitivity analyses (Supplemental Figure S3).

#### 3.3.3. Diagnostic Performance of Neuroimaging Markers of CSVD and the APOE ε4 Allele for Cognitive Outcomes

Although the primary aim of this meta-analysis was to evaluate associations between CSVD markers and cognitive outcomes, the diagnostic performance of these imaging and genetic markers was also assessed. This exploratory analysis provides insight into their clinical utility for identifying patients at risk of cognitive decline. Data were available for all markers and cognitive outcome subtypes, with the exception of WMHs in relation to VaD and AD, where too few studies precluded pooled estimates.

Diagnostic performance was modest. Across markers, sensitivities were consistently low, ranging from 22% (95% CI: 0.13–0.35) for CMBs predicting AD to 50% (95% CI: 0.25–0.75) for CMBs predicting VaD. Specificities were generally moderate-to-high, spanning 66% (95% CI: 0.38–0.86) for WMHs with MCI to 90% (95% CI: 0.61–0.98) for lacunes with ACD. This pattern indicates that these markers are more reliable for ruling out cognitive impairment in unaffected individuals than for accurately identifying those at risk.

The APOE ε4 allele displayed similar limitations. Its sensitivity for predicting GCI was only 35% (95% CI: 0.29–0.41), whereas specificity was moderate at 76% (95% CI: 0.73–0.79). These values suggest that while APOE ε4 positivity raises the likelihood of cognitive decline, a large proportion of affected patients will not be captured by genetic screening alone.

Discriminatory ability, as assessed by AUROC, was also modest across most markers. AUROC values ranged from poor (0.44; 95% CI: 0.40–0.49 for lacunes with MCI) to moderate (0.81; 95% CI: 0.78–0.85 for CMBs with VaD). These results imply that while some combinations of markers and outcomes (such as CMBs with VaD) demonstrate reasonable discriminative potential, most fall short of thresholds typically considered clinically actionable. The full set of pooled sensitivities, specificities, likelihood ratios, and AUROC values is provided in Supplemental Table S5, with SROC curves shown in Supplemental Figures S1 and S2.

#### 3.3.4. Certainty of Evidence (NEUROGEN-SVD)

The certainty of evidence across NEUROGEN-SVD outcomes ranged from low to moderate. Evidence supporting the association between WMHs and VaD was rated moderate certainty, reflecting a very strong effect size (OR 10.35) and low heterogeneity, despite variability in diagnostic criteria. Associations between WMHs and AD, as well as lacunes with GCI, were rated low certainty due to the heterogeneity and indirectness of outcome definitions. The APOE ε4–GCI association was also rated low certainty, reflecting inconsistency across populations. The NEUROGEN-SVD Summary of Findings (Table 7) provides pooled effect sizes, absolute risk differences, and certainty ratings for each outcome.

## 4. Discussion

This NEUROGEN-SVD study offers one of the most comprehensive syntheses to date on the relationship between CSVD, genetic susceptibility, and cognitive outcomes. Drawing on data from nearly 18,500 participants across 39 studies, the meta-analysis demonstrates that CSVD is a major driver of cognitive decline, extending beyond VaD to encompass the full spectrum of impairment, from MCI to ACD and AD. These findings reinforce CSVD as a central determinant of late-life cognitive trajectories and highlight its dual role in both vascular and neurodegenerative pathways.

While prior meta-analyses have largely focused on the prevalence of VaD, with estimates ranging between 36 and 67% depending on diagnostic criteria [57,58], few have examined GCI and MCI more broadly within CSVD populations. The current meta-analysis advances this field by estimating the pooled prevalence of GCI at 57% (95% CI: 51–62%), which is notably higher than a previous estimate of 44.1% [33]. It also reports an MCI prevalence of 46%, underscoring that CSVD is not only a driver of late-stage dementia but is also prominently involved in early cognitive decline. The relatively consistent prevalence of MCI across regions reinforces its potential as a stable and clinically meaningful early marker. The higher heterogeneity in GCI prevalence likely reflects the broad and variable definitions of GCI, which encompass a spectrum from MCI through to overt dementia.

These findings support an evolving model of CSVD-related GCI as a continuum. Rather than being defined solely by advanced pathology, CSVD contributes to subtle, microstructural tissue damage that disrupts connectivity long before overt lesion burden is visible [59]. This highlights opportunities for early intervention through aggressive management of vascular risk factors, lifestyle modification, and cognitive rehabilitation before cognitive deterioration becomes irreversible.

Previous meta-analyses have tended to examine individual imaging markers or their associations with specific cognitive domains [60] or dementia [61,62,63] outcomes. Based on current understanding, this is the first meta-analysis to systematically compare multiple CSVD markers (WMHs, CMBs, and lacunes) and genetic risk (APOE ε4) across a range of cognitive outcomes, from MCI through ACD, VaD, AD, and GCI. These results show that associations were generally stronger for later stages of impairment (ACD, VaD, and AD) than for MCI. This gradient is consistent with the progressive nature of CSVD, in which the cumulative burden of lesions disrupts white matter tracts and cortical–subcortical connectivity, gradually leading to widespread network dysfunction and cognitive impairment [59,64]. Weaker associations observed with MCI likely reflect that lesion burden in early stages may not have crossed the threshold needed to cause a clinically detectable decline.

Among individual markers, WMHs demonstrated the strongest and most consistent associations with cognitive outcomes, particularly VaD, where odds were increased more than tenfold. These findings align with prior evidence and reinforce the view of WMHs as markers of diffuse ischemia from chronic microvascular compromise, directly linked to sustained cerebral hypoperfusion [2,65]. Lacunes also showed significant associations across all outcomes, though with greater heterogeneity, likely reflecting variations in imaging protocols and challenges in distinguishing them from perivascular spaces [16]. CMBs demonstrated weaker associations overall, though they became significant in dementia outcomes, suggesting their cognitive impact becomes apparent only once a threshold burden is reached [66].

The APOE ε4 allele was significantly associated with GCI, extending its relevance beyond AD risk alone. Emerging evidence suggests that APOE ε4 may exacerbate vascular injury through mechanisms such as neuroinflammation [67,68] and blood–brain barrier (BBB) dysfunction, independent of amyloid-β [69]. These observations support the Integrated Vascular–Neurodegenerative Continuum Hypothesis [2], which posits that vascular and neurodegenerative processes converge through overlapping pathways, lowering the threshold for GCI. This concept is reinforced by the observed associations between CSVD markers and AD, as well as by the influence of APOE ε4 across multiple cognitive subtypes. White matter lesions in AD are themselves heterogeneous: while many reflect chronic hypoperfusion [70] or amyloid angiopathy [71], others—particularly posterior periventricular lesions [72]—may arise from primary neurodegenerative mechanisms such as tau-mediated axonal degeneration [73,74,75]. Recognizing these dual etiologies is essential for interpreting the burden of WMHs in mixed dementias. Related biomarkers, including soluble low-density lipoprotein receptor-related protein-1 (sLRP-1), have been implicated in both vascular [76] and amyloidogenic cascades [77]; however, current data do not support sLRP-1 as a CSVD-specific marker distinct from AD or other comorbid conditions.

In addition to associations, the diagnostic performance of these markers was explored. Across neuroimaging and genetic measures, sensitivities were uniformly low, specificities were moderate-to-high, and AUROC values were modest. This indicates that while these markers have diagnostic weight when present, they lack sufficient sensitivity to serve as standalone screening tools. The findings underscore that cognitive decline in CSVD is unlikely to arise from isolated vascular or neurodegenerative processes but rather from their overlapping contributions [2]. This has two major implications. First, early systematic screening for GCI in CSVD populations is warranted, particularly targeting MCI, which appears as a consistent and robust early marker. Second, multimodal diagnostic strategies, integrating imaging markers, genetic risk, vascular burden, and clinical data, are needed to enhance diagnostic accuracy and risk stratification. Emerging BBB-permeability imaging, including arterial-spin labeling (ASL) [78], dynamic contrast-enhanced MRI [79], and novel PET tracers [80], provides complementary mechanistic insight into microvascular injury. Incorporating BBB metrics with WMHs and APOE status may refine early CSVD detection [81]. For Asian populations, where prevalence is higher, aggressive management of hypertension and diabetes may have particularly strong preventive effects.

High heterogeneity was observed across several outcomes, especially for associations involving GCI and AD with CMBs and lacunes. This likely reflects variation in imaging protocols, definitions of markers, diagnostic criteria, and study populations, which spanned hospital-based cohorts to community samples. Differences in adjustment for confounders and inter-rater reliability further contributed to variability. Funnel plots and formal bias tests suggested some small-study effects, particularly for lacunes and APOE ε4, though the overall direction of associations was consistent in sensitivity analyses. These observations highlight the pressing need for standardized imaging protocols, operational definitions of CSVD markers, and harmonized neuropsychological assessments to improve comparability and reduce heterogeneity. Insights from monogenic CSVD syndromes such as CADASIL (NOTCH3 mutations) [82,83] and COL4A1/2-related angiopathies [84] illuminate pure vascular pathways leading to cognitive impairment. Observations from these disorders, where lesion burden predicts cognitive decline independent of classical risk factors [85,86], reinforce the vascular mechanisms highlighted in our meta-analysis.

While this meta-analysis demonstrates robust associations between CSVD markers and cognitive outcomes, it is equally important to assess the certainty of these findings. Using the GRADE framework within NEUROGEN-SVD, the strengths and limitations of the available evidence were systematically evaluated. Certainty of evidence ranged from low to moderate, with the strongest confidence observed for WMHs as predictors of VaD, and lower confidence for associations with GCI and AD due to heterogeneity, diagnostic variability, and potential publication bias (Table 7). The NEUROGEN-SVD GRADE assessment therefore underscores both the strengths and gaps in the current evidence base. The most consistent and reliable signal was the strong association between WMHs and VaD, supported by moderate-certainty evidence, reinforcing WMHs as a key imaging marker of vascular cognitive outcomes. Associations with GCI and AD were downgraded to low certainty, reflecting variation in diagnostic criteria, imaging protocols, and study designs. These findings emphasize two key points: first, the directional consistency of associations across multiple markers suggests a genuine biological link between CSVD pathology and cognitive decline; second, the overall low-to-moderate certainty highlights the urgent need for harmonized definitions, standardized imaging protocols, and longitudinal multimodal studies. Incorporating lesion severity quantification, advanced neuroimaging, and genetic–vascular interaction models within the NEUROGEN-SVD framework will be critical for improving evidence certainty and advancing precision medicine strategies for CSVD-related cognitive impairment. Given the predominantly cross-sectional evidence, future multicenter cohorts with serial imaging and harmonized cognitive batteries are essential to track the transition from MCI to dementia, validate temporal associations, and test whether early multimodal risk scores predict trajectory.

### Limitations

This meta-analysis has several limitations. First, pooled estimates could only be derived for WMHs, CMBs, and lacunes; data on other CSVD markers, such as cortical superficial siderosis and perivascular spaces, were insufficient. Second, most studies reported presence/absence rather than severity or burden of lesions, limiting the ability to assess dose–response relationships. Third, the analysis of APOE ε4 was restricted to GCI, as too few studies reported across other cognitive outcomes. Fourth, most included studies were cross-sectional, restricting insights into progression from MCI to dementia. Fifth, it was impossible to evaluate the combined predictive value of imaging and genetic markers simultaneously. Sixth, although limited exploratory subgroup analyses were performed, detailed stratified subgroup analyses and meta-regression could not be undertaken due to insufficient stratified reporting. No data imputation was attempted. Finally, substantial heterogeneity across studies likely influenced the precision and reliability of pooled estimates.

## 5. Conclusions

The NEUROGEN-SVD study provides one of the most comprehensive evidence syntheses to date linking CSVD imaging and genetic markers with cognitive outcomes. This meta-analysis confirms that WMHs, lacunes, CMBs, and the APOE ε4 allele are significantly associated with both early (MCI and GCI) and late (ACD, VaD, and AD) stages of cognitive impairment, reinforcing the concept of a vascular–neurodegenerative continuum. Certainty of evidence ranged from low to moderate, with the strongest confidence in WMHs as a predictor of vascular dementia. These findings highlight the urgent need for harmonized diagnostic frameworks, standardized imaging protocols, and longitudinal multimodal studies within NEUROGEN-SVD and related initiatives. Strengthening the certainty of evidence will be critical to advancing precision strategies for early detection, risk stratification, and prevention of CSVD-related cognitive decline.

## Figures and Tables

**Table 4:** Pooled prevalence of cognitive outcomes in CSVD patients: summary effects and heterogeneity.

Subgroup	Studies (N)	Participants (*n*)	Summary Effects					Heterogeneity			
			Crude Prevalence	Pooled Prevalence	95% CI	z	*p*	Q	I^2^ (%)	*p* (Q)	*τ* ^2^
Overall	16	2118	53.5%	53%	0.49–0.58	33.88	*p* < 0.001	62.89	76.15	*p* < 0.001	0.02
GCI	10	1518	55.9%	57%	0.51–0.62	28.48	*p* < 0.001	42.15	78.65	*p* < 0.001	0.02
MCI	7	766	46.3%	46%	0.42–0.51	31.02	*p* < 0.001	8.91	32.67	*p* = 0.18	0.00

Abbreviations: ACI: all-cause dementia; GCI: global cognitive impairment; MCI: mild cognitive impairment; 95% CI: 95% confidence interval; CSVD: cerebral small vessel disease. Notes: (1) “Crude prevalence” is the unweighted proportion; “Pooled prevalence” is the random-effects estimate; (2) Random-effects meta-analysis used the DerSimonian–Laird (DL) method; (3) Heterogeneity: Q (Cochran’s Q, χ^2^ with df = N − 1); I^2^ (% between-study variability); τ^2^ (between-study variance, DerSimonian–Laird); (4) z and *p* are two-sided tests of the pooled effect; (5) One study with overlapping cohorts across MCI and GCI was excluded from the overall pooled estimate.

**Table 5:** Associations between CSVD imaging/genetic markers and cognitive outcomes: results by marker/genetic factor.

Imaging/Genetic Marker	Cognitive Subgroup	Studies (N)	Participants (*n*)	Summary Effects DL		Heterogeneity				
				Pooled OR (95% CI)	*p*, z	Cochran’s Q	*p* (Q)	H	I^2^ (%)	*τ* ^2^
CMBs	MCI	8	2301	1.93 [1.48; 2.51]	*p* < 0.001, z = 4.813	11.11	0.134	1.260	37.0	0.051
	GCI	5	1505	1.70 [0.92; 3.16]	*p* = 0.091, z = 1.689	13.45	0.009	1.834	70.3	0.310
	ACD	9	5987	1.92 [1.41; 2.60]	*p* < 0.001, z = 4.20	15.75	0.046	1.403	49.2	0.101
	VaD	5	4399	4.70 [2.10; 10.52]	*p* < 0.001, z = 3.77	11.49	0.022	1.695	65.2	0.535
	AD	7	5369	1.52 [1.04; 2.24]	*p* = 0.033, z = 2.14	12.40	0.054	1.437	80.8	2.275
WMHs	MCI	4	3513	2.42 [1.57; 3.74]	*p* < 0.001, z = 3.980	5.86	0.118	1.398	48.8	0.094
	VaD	3	1815	10.35 [7.32; 14.64]	*p* < 0.001, z = 13.229	1.90	0.388	0.974	0.0	0.000
	AD	3	2621	2.78 [1.27; 6.09]	*p* = 0.011, z = 2.558	12.98	0.002	2.548	84.6	0.394
Lacunes	MCI	4	1836	2.70 [1.25; 5.84]	*p* = 0.011, z = 2.528	14.40	0.002	2.191	79.2	0.398
	GCI	5	1505	2.41 [1.33; 4.40]	*p* = 0.004, z = 2.88	10.07	0.039	1.586	60.3	0.246
	ACD	4	1440	3.18 [1.24; 8.20]	*p* = 0.017, z = 2.40	20.51	0.000	2.615	85.4	0.664
APOE ε4	GCI	6	2398	1.80 [1.41, 2.29]	*p* < 0.001, z = 4.729	6.13	0.294	1.107	68.3	0.017

Abbreviations: ACD: all-cause dementia; AD: Alzheimer’s disease; APOE: apolipoprotein E; CMBs: cerebral microbleeds; GCI: global cognitive impairment; MCI: mild cognitive impairment; OR: odds ratios; VaD: vascular dementia; WMHs: white matter hyperintensities; 95% CI: 95% confidence interval; df: degrees of freedom. Notes: (1) Model: random-effects DerSimonian-Laird (DL); (2) Effect size is pooled OR (95% CI); z and *p* test log(OR) = 0 (two-sided); (3) Heterogeneity: Q (Cochran’s Q, χ^2^ with df = N − 1); H (√[Q/df]); I^2^ (%); τ^2^ (DL); (4) For N ≤ 3, interpret I^2^/H cautiously due to low df; (5) When a study reports multiple cognitive outcomes, each is listed separately and analysed as an independent comparison.

**Table 6:** Associations between CSVD imaging/genetic markers and cognitive outcomes: results by cognitive outcome.

Cognitive Outcome	Marker	Studies (N)	Participants (*n*)	Summary Effects DL		Heterogeneity				
				Pooled OR (95% CI)	*p*, z	Cochran’s Q	*p* (Q)	H	I^2^	*τ* ^2^
MCI	CMB	8	2301	1.93 [1.48; 2.51]	*p* < 0.001, z = 4.813	11.11	0.134	1.260	37.0	0.051
	WMHs	4	3513	2.42 [1.57; 3.74]	*p* < 0.001, z = 3.980	5.86	0.118	1.398	48.8	0.094
	Lacunes	4	1836	2.70 [1.25; 5.84]	*p* = 0.011, z = 2.528	14.40	0.002	2.191	79.2	0.398
GCI	CMBs	5	1505	1.70 [0.92; 3.16]	*p* = 0.091, z = 1.689	13.45	0.009	1.834	70.3	0.310
	Lacunes	5	1505	2.41 [1.33; 4.40]	*p* = 0.004, z = 2.88	10.07	0.039	1.586	60.3	0.246
	APOE ε4 allele	6	2398	1.80 [1.41, 2.29]	*p* < 0.001, z = 4.729	6.13	0.294	1.107	68.3	0.017
ACD	CMBs	9	5987	1.92 [1.41; 2.60]	*p* < 0.001, z = 4.20	15.75	0.046	1.403	49.2	0.101
	Lacunes	4	1440	3.18 [1.24; 8.20]	*p* = 0.017, z = 2.40	20.51	0.000	2.615	85.4	0.664
VaD	CMBs	5	4399	4.70 [2.10; 10.52]	*p* < 0.001, z = 3.77	11.49	0.022	1.695	65.2	0.535
	WMHs	3	1815	10.35 [7.32; 14.64]	*p* < 0.001, z = 13.229	1.90	0.388	0.974	0.0	0.000
AD	CMBs	7	5369	1.52 [1.04; 2.24]	*p* = 0.033, z = 2.14	12.40	0.054	1.437	80.8	2.275
	WMHs	3	2621	2.78 [1.27; 6.09]	*p* = 0.011, z = 2.558	12.98	0.002	2.548	84.6	0.394

Abbreviations: ACD: all-cause dementia; AD: Alzheimer’s disease; APOE: apolipoprotein E; CMBs: cerebral microbleeds; GCI: global cognitive impairment; MCI: mild cognitive impairment; OR: odds ratios; VaD: vascular dementia; WMHs: white matter hyperintensities; 95% CI: 95% confidence interval; df: degrees of freedom. Notes: (1) Model: random-effects, DerSimonian and Laird (DL); (2) Pooled OR (95% CI) per marker within each outcome; z and *p* test log(OR) = 0 (two-sided); (3) Heterogeneity: Q (Cochran’s Q, χ^2^ with df = N − 1); H (√[Q/df]); I^2^ (%); τ^2^ (between-study variance); (4) Studies reporting multiple markers/outcomes appear as separate rows and are analysed as independent comparisons.

**Table 7:** NEUROGEN-SVD GRADE summary of findings: certainty of evidence for CSVD imaging and genetic markers in cognitive outcomes.

Outcome	No. of Studies (Participants)	Study Design	Relative Effect (95% CI)	Assumed Risk (Control)	Risk with Marker	Absolute Effect (per 1000)	Certainty of Evidence	Reasons
CMBs → MCI	8 (~2301)	Observational (meta-analysis, random-effects)	OR 1.93 (1.48–2.51)	300 per 1000	450 per 1000	150 more per 1000	⊕⊕◯◯ Low	−1 risk of bias, −1 inconsistency, +1 moderate effect
CMBs → ACD	9 (~5987)	Observational (meta-analysis, random-effects)	OR 1.92 (1.41–2.60)	350 per 1000	500 per 1000	150 more per 1000	⊕⊕⊕◯ Moderate	−1 risk of bias, +1 consistent effect
CMBs → VaD	5 (~4399)	Observational (meta-analysis, random-effects)	OR 4.70 (2.10–10.52)	200 per 1000	560 per 1000	360 more per 1000	⊕⊕◯◯ Low to Moderate	−1 heterogeneity, +1 strong effect
CMBs → AD	7 (~5369)	Observational (meta-analysis, random-effects)	OR 1.52 (1.04–2.24)	250 per 1000	360 per 1000	110 more per 1000	⊕⊕◯◯ Low	−1 imprecision, −1 heterogeneity
WMHs → MCI	4 (~3513)	Observational (meta-analysis, random-effects)	OR 2.42 (1.57–3.74)	300 per 1000	520 per 1000	220 more per 1000	⊕⊕◯◯ Low to Moderate	−1 risk of bias, −1 inconsistency, +1 effect size
WMHs → VaD	4 (~1815)	Observational (meta-analysis, random-effects)	OR 10.35 (7.32–14.64)	200 per 1000	740 per 1000	540 more per 1000	⊕⊕⊕◯ Moderate	−1 diagnostic variability, +1 very strong effect, +1 low heterogeneity
WMHs → AD	3 (~2621)	Observational (meta-analysis, random-effects)	OR 2.78 (1.27–6.09)	250 per 1000	480 per 1000	230 more per 1000	⊕⊕◯◯ Low	−1 inconsistency, −1 indirectness, −1 imprecision
Lacunes → MCI	4 (~1836)	Observational (meta-analysis, random-effects)	OR 2.70 (1.25–5.84)	300 per 1000	560 per 1000	260 more per 1000	⊕⊕◯◯ Low	−1 heterogeneity, −1 indirectness
Lacunes → GCI	5 (~1505)	Observational (meta-analysis, random-effects)	OR 2.41 (1.33–4.40)	400 per 1000	610 per 1000	210 more per 1000	⊕⊕◯◯ Low	−1 marker misclassification, −1 heterogeneity
Lacunes → ACD	4 (~1440)	Observational (meta-analysis, random-effects)	OR 3.18 (1.24–8.20)	350 per 1000	640 per 1000	290 more per 1000	⊕⊕◯◯ Low	−1 inconsistency, −1 imprecision
APOE ε4 → GCI	6 (~2398)	Observational (meta-analysis, random-effects)	OR 1.80 (1.41–2.29)	400 per 1000	570 per 1000	170 more per 1000	⊕⊕◯◯ Low	−1 inconsistency, −1 indirectness

Assumed risk (control) represents the baseline probability of the outcome in patients without the CSVD marker or APOE ε4 allele, expressed per 1000 individuals. This value was derived from the median or pooled control-group risk across included studies. Risk with the marker was calculated by converting the pooled odds ratio (OR) into an absolute risk using the following formula:
(1) $$R_{1}=\frac{OR\times R_{0}}{1-R_{0}+(OR\times R_{0})}$$
where $R_{0}$ is the assumed baseline risk. Absolute effect is the difference between the risk with marker and the assumed risk, expressed as the number of additional (or fewer) events per 1000 individuals. Confidence intervals for absolute effects were derived by applying the same conversion using the lower and upper bounds of the pooled OR. Abbreviations: NEUROGEN-SVD = Neuroimaging and Genetic Markers in Small Vessel Disease study; GCI = global cognitive impairment; MCI = mild cognitive impairment; ACD = all-cause dementia; VaD = vascular dementia; AD = Alzheimer’s disease; WMHs = white matter hyperintensities; CMBs = cerebral microbleeds; OR = odds ratio. GRADE Working Group grades of evidence: ⊕⊕⊕⊕ High: Very confident that the true effect lies close to the estimate; ⊕⊕⊕◯ Moderate: Moderately confident; true effect likely close but may differ; ⊕⊕◯◯ Low: Limited confidence; true effect may differ substantially; ⊕◯◯◯ Very low: Very little confidence; true effect likely substantially different.

## Data Availability

The original contributions presented in this study are included in the article/Supplementary Material. Further inquiries can be directed to the corresponding author.

## Supplementary Materials

The following supporting information can be downloaded at: https://www.mdpi.com/article/10.3390/diagnostics15202585/s1, Table S1: PRISMA-2020 Checklist; Table S2: MOOSE Checklist; Table S3: Modified Jadad Analysis for Methodological Quality; Table S4: Funding Bias Scores for Studies; Table S5: Summary Data and Performance Estimates for Meta-analysis on the Diagnostic Accuracy of CSVD Neuroimaging Markers and APOE ε4 Allele Carrier Status and Cognitive Outcomes; Table S6: Outputs from Egger’s Regression Test; Table S7: Outputs from Deek’s Funnel-Plot Asymmetry Test; Figure S1: Summary Receiver Operating Characteristic (SROC) Curves for Imaging Markers of CSVD and APOE ε4 Allele Carrier Status (1); Figure S2: Summary Receiver Operating Characteristic (SROC) Curves for Imaging Markers of CSVD and APOE ε4 Allele Carrier Status (2); Figure S3: Sensitivity Analysis on the Association between Imaging and Genetic Markers of CSVD and Cognitive Outcomes; Figure S4: Graphs of Egger’s Regression Test; Figure S5: Graphs of Funnel Plots; Figure S6: Graphs of Deek’s Funnel-Plot Asymmetry Test; Figure S7: Graphs of Fagan’s Normogram. References [87,88] are cited in the Supplementary Materials.
