# Supplementary material for: Neuroimaging and Genetic Markers of Cerebral Small Vessel Disease and Cognitive Outcomes: A Systematic Review and Meta-Analysis (NEUROGEN-SVD Study)
Source: Diagnostics (Basel). 2025 Oct 13;15(20):2585. doi: 10.3390/diagnostics15202585 (PMC12563764; doi:10.3390/diagnostics15202585)

SUPPLEMENTAL INFORMATION

Neuroimaging and Genetic Markers of Cerebral Small Vessel Disease and Cognitive Outcomes: A Systematic Review and Meta-Analysis (NEUROGEN-SVD Study)

Chelsea Jin, Roy G. Beran and Sonu M. M. Bhaskar*

*Correspondence to: Dr Sonu M. M. Bhaskar, MD, PhD, FANA

Email: Sonu.Bhaskar@globalhealthneurolab.org

List of Contents

1. **Search Strategy (Keywords/MeSH Terms)**
    - **a. PubMed Search Strategy**
    - **b. Embase Search Strategy**
    - **c. Scopus Search Strategy**
    - **d. Web of Science Search Strategy**
    - **e. Cochrane Library Search Strategy**
2. **Supplemental Tables**
    - **a. Supplemental Table S1:** PRISMA-2020 Checklist
    - **b. Supplemental Table S2:** MOOSE Checklist
    - **c. Supplemental Table S3:** Modified Jadad Analysis for Methodological Quality
    - **d. Supplemental Table S4:** Funding Bias Scores for Studies
    - **e. Supplemental Table S5:** Summary Data and Performance Estimates for Meta-analysis on the Diagnostic Accuracy of CSVD Neuroimaging Markers and APOE ε4 Allele Carrier Status and Cognitive Outcomes
    - **f. Supplemental Table S6:** Outputs from Egger's Regression Test
    - **g. Supplemental Table S7:** Outputs from Deek's Funnel-Plot Asymmetry Test
3. **Supplemental Figures**
    - **a. Supplemental Figure S1:** Summary Receiver Operating Characteristic (SROC) Curves for Imaging Markers of CSVD and APOE ε4 Allele Carrier Status (1)
    - **b. Supplemental Figure S2:** Summary Receiver Operating Characteristic (SROC) Curves for Imaging Markers of CSVD and APOE ε4 Allele Carrier Status (2)
    - **c. Supplemental Figure S3:** Sensitivity Analysis on the Association between Imaging and Genetic Markers of CSVD and Cognitive Outcomes
    - **d. Supplemental Figure S4:** Graphs of Egger's Regression Test
    - **e. Supplemental Figure S5:** Graphs of Funnel Plots
    - **f. Supplemental Figure S6:** Graphs of Deek's Funnel-Plot Asymmetry Test
    - **g. Supplemental Figure S7:** Graphs of Fagan's Normogram

1. **Search Strategy (Keywords/MeSH Terms)**

a. PubMed Search Strategy

#1: ("Magnetic Resonance Imaging"[Mesh] OR "MRI"[tiab] OR "Brain MRI"[tiab]) OR ("Neuroimaging"[Mesh] OR "Brain Imaging"[tiab] OR "Cerebral Imaging"[tiab] OR "Structural Imaging"[tiab]) OR ("Diffusion Tensor Imaging"[Mesh] OR "DTI"[tiab] OR "White Matter Imaging"[tiab]) OR ("Susceptibility-Weighted Imaging"[tiab] OR "SWI"[tiab]) OR ("Fluid-Attenuated Inversion Recovery"[tiab] OR "FLAIR"[tiab]) OR ("T2-weighted imaging"[tiab] OR "T1-weighted imaging"[tiab]) OR ("Positron Emission Tomography"[Mesh] OR "PET Imaging"[tiab]) OR ("Tomography, X-Ray Computed"[Mesh] OR "CT Scan"[tiab] OR "CT Imaging"[tiab])

#2: ("Genetic Predisposition to Disease"[Mesh] OR "Genetic Risk"[tiab] OR "Genetic Variants"[tiab] OR "Genome-Wide Association Study"[Mesh] OR "GWAS"[tiab]) OR ("APOE"[tiab] OR "Apolipoproteins E"[Mesh] OR "APOE ε4"[tiab]) OR ("CADASIL"[Mesh] OR "NOTCH3"[tiab]) OR ("COL4A1"[tiab] OR "COL4A2"[tiab]) OR ("HTRA1"[tiab] OR "CARASIL"[tiab]) OR ("ABCC6"[tiab] OR "CRPPA"[tiab] OR "LAMA2"[tiab]) OR ("MTHFR"[tiab] OR "Homocysteine"[tiab])

#3: ("Cerebral Small Vessel Diseases"[Mesh] OR "Small Vessel Disease"[tiab] OR "Cerebral Microangiopathy"[tiab] OR "White Matter Hyperintensities"[tiab] OR "Lacunar Infarcts"[tiab] OR "Perivascular Spaces"[tiab] OR "Microbleeds"[tiab] OR "Leukoaraiosis"[tiab])

#4: ("Dementia"[Mesh] OR "Cognitive Dysfunction"[Mesh] OR "Dementia, Vascular"[Mesh] OR "Subcortical Ischemic Vascular Dementia"[tiab] OR "Cognitive Impairment"[tiab] OR "Cognitive Decline"[tiab] OR "Vascular Cognitive Impairment"[tiab])

Search Query: (#1 OR #2) AND #3 AND #4

Filters applied: Adaptive Clinical Trial, Clinical Trial, Comparative Study, Controlled Clinical Trial, Meta-Analysis, Multicenter Study, Observational Study, Randomized Controlled Trial, Systematic Review, English, Humans, Adult: 19+ years, from 2005

Results: 356

b. Embase Search Strategy

#1: ('nuclear magnetic resonance imaging' or 'MRI' or 'Brain MRI' or ('Neuroimaging' or 'Brain Imaging' or 'Cerebral Imaging' or 'Structural Imaging') or ('Diffusion Tensor Imaging' or 'DTI' or 'White Matter Imaging') or ('Susceptibility Weighted Imaging' or 'SWI') or ('Fluid-Attenuated Inversion Recovery Imaging' or 'FLAIR') or ('T2-weighted imaging' or 'T-1-weighted imaging') or ('Positron Emission Tomography' or 'PET Imaging') or ('Computer Assisted Tomography' or 'CT SCAN' or 'CT imaging')).ti,ab. and (systematic review/ or meta analysis/ or randomized controlled trial/ or comparative study/ or case control study/ or controlled clinical trial/ or clinical trial/)

#2: ('Genetic Predisposition' or 'Genetic Risk' or 'Genetic Variants' or 'Genome-Wide Association Study' or 'GWAS' or ('APOE' or 'Apolipoprotein E' or 'APO E4') or ('CADASIL' or 'NOTCH3') or ('COL4A1' or 'COL4A2') or ('HTRA1' or 'CARASIL') or ('ABCC6' or 'CRPPA' or 'LAMA2') or ('MTHFR' or

'Homocysteine')).ti,ab. and (systematic review/ or meta analysis/ or randomized controlled trial/ or comparative study/ or case control study/ or controlled clinical trial/ or clinical trial/)

#3: ("Cerebral Small Vessel Diseases" or "Small Vessel Disease" or "Cerebral Microangiopathy" or "White Matter Hyperintensities" or "Lacunar Infarcts" or "Perivascular Spaces" or "Microbleeds" or "Leukoaraiosis").ti,ab.
and (systematic review/ or meta analysis/ or randomized controlled trial/ or comparative study/ or case control study/ or controlled clinical trial/ or clinical trial/)

#4: ("Dementia" or "Cognitive Dysfunction" or "Vascular Dementia" or "Subcortical Ischemic Vascular Dementia" or "Cognitive Impairment" or "Cognitive Decline" or "Vascular Cognitive Impairment").ti,ab.and (systematic review/ or meta analysis/ or randomized controlled trial/ or comparative study/ or case control study/ or controlled clinical trial/ or clinical trial/)

Search Query: (#1 OR #2) AND #3 AND #4

Filters applied: human and english language and yr="2005-Current" and (adult <18 to 64 years> or aged <65+ years>)

Results: 381

c. Scopus Search Strategy

#1: TITLE-ABS(("nuclear magnetic resonance imaging" OR "MRI" OR "Brain MRI") OR ("Neuroimaging" OR "Brain Imaging" OR "Cerebral Imaging" OR "Structural Imaging") OR ("Diffusion Tensor Imaging" OR "DTI" OR "White Matter Imaging") OR ("Susceptibility Weighted Imaging" OR "SWI") OR ("Fluid-Attenuated Inversion Recovery Imaging" OR "FLAIR") OR ("T2-weighted imaging" OR "T-1-weighted imaging") OR ("Positron Emission Tomography" OR "PET Imaging") OR ("Computer Assisted Tomography" OR "CT SCAN" OR "CT imaging"))

#2: TITLE-ABS(("Genetic Predisposition to Disease" OR "Genetic Risk" OR "Genetic Variants" OR "Genome-Wide Association Study" OR "GWAS" OR "APOE" OR "Apolipoprotein E" OR "APOE ε4" OR "CADASIL" OR "NOTCH3" OR "COL4A1" OR "COL4A2" OR "HTRA1" OR "CARASIL" OR "ABCC6" OR "CRPPA" OR "LAMA2" OR "MTHFR" OR "Homocysteine"))

#3: TITLE-ABS("Cerebral Small Vessel Diseases" OR "Small Vessel Disease" OR "Cerebral Microangiopathy" OR "White Hyperintensities" OR "Lacunar Infarcts" OR "Perivascular Spaces" OR "Microbleeds" OR "Leukoaraiosis")

#4: TITLE-ABS("Dementia" OR "Cognitive Dysfunction" OR "Vascular Dementia" OR "Subcortical Ischemic Vascular Dementia" OR "Cognitive Impairment" OR "Cognitive Decline" OR "Vascular Cognitive Impairment")

Search Query: #1 AND #2 AND #3 AND #4

Filters: Humans, English, 2005-2025, Article, Review, Final Publication Stage, Journal

Results: 1880

d. Web of Science Search Strategy

#1: (TI=(("nuclear magnetic resonance imaging" OR "MRI" OR "Brain MRI") OR ("Neuroimaging" OR "Brain Imaging" OR "Cerebral Imaging" OR "Structural Imaging") OR ("Diffusion Tensor Imaging" OR "DTI" OR "White Matter Imaging") OR ("Susceptibility Weighted Imaging" OR "SWI") OR ("Fluid-Attenuated Inversion Recovery Imaging" OR "FLAIR") OR ("T2-weighted imaging" OR "T-1-weighted imaging") OR ("Positron Emission Tomography" OR "PET Imaging") OR ("Computer Assisted Tomography" OR "CT SCAN" OR "CT imaging")) OR AB=(("nuclear magnetic resonance imaging" OR "MRI" OR "Brain MRI") OR ("Neuroimaging" OR "Brain Imaging" OR "Cerebral Imaging" OR "Structural Imaging") OR ("Diffusion Tensor Imaging" OR "DTI" OR "White Matter Imaging") OR ("Susceptibility Weighted Imaging" OR "SWI") OR ("Fluid-Attenuated Inversion Recovery Imaging" OR "FLAIR") OR ("T2-weighted imaging" OR "T-1-weighted imaging") OR ("Positron Emission Tomography" OR "PET Imaging") OR ("Computer Assisted Tomography" OR "CT SCAN" OR "CT imaging")))

#2: (TI=("Genetic Predisposition to Disease" OR "Genetic Risk" OR "Genetic Variants" OR "Genome-Wide Association Study" OR "GWAS" OR "APOE" OR "Apolipoprotein E" OR "APOE ε4" OR "CADASIL" OR "NOTCH3" OR "COL4A1" OR "COL4A2" OR "HTRA1" OR "CARASIL" OR "ABCC6" OR "CRPPA" OR "LAMA2" OR "MTHFR" OR "Homocysteine") OR AB=("Genetic Predisposition to Disease" OR "Genetic Risk" OR "Genetic Variants" OR "Genome-Wide Association Study" OR "GWAS" OR "APOE" OR "Apolipoprotein E" OR "APOE ε4" OR "CADASIL" OR "NOTCH3" OR "COL4A1" OR "COL4A2" OR "HTRA1" OR "CARASIL" OR "ABCC6" OR "CRPPA" OR "LAMA2" OR "MTHFR" OR "Homocysteine"))

#3: (TI=("Cerebral Small Vessel Diseases" OR "Small Vessel Disease" OR "Cerebral Microangiopathy" OR "White Matter Hyperintensities" OR "Lacunar Infarcts" OR "Perivascular Spaces" OR "Microbleeds" OR "Leukoaraiosis") OR AB=("Cerebral Small Vessel Diseases" OR "Small Vessel Disease" OR "Cerebral Microangiopathy" OR "White Matter Hyperintensities" OR "Lacunar Infarcts" OR "Perivascular Spaces" OR "Microbleeds" OR "Leukoaraiosis"))

#4: (TI=("Dementia" OR "Cognitive Dysfunction" OR "Vascular Dementia" OR "Subcortical Ischemic Vascular Dementia" OR "Cognitive Impairment" OR "Cognitive Decline" OR "Vascular Cognitive Impairment") OR AB=("Dementia" OR "Cognitive Dysfunction" OR "Vascular Dementia" OR "Subcortical Ischemic Vascular Dementia" OR "Cognitive Impairment" OR "Cognitive Decline" OR "Vascular Cognitive Impairment"))

Search Query: (#1 OR #2) AND #3 AND #4

Filters applied: (PY==("2005" OR "2025" OR "2024" OR "2023" OR "2022" OR "2021" OR "2020" OR "2019" OR "2018" OR "2017" OR "2016" OR "2015" OR "2014" OR "2013" OR "2012" OR "2011" OR "2010" OR "2009" OR "2008" OR "2007" OR "2006") AND DT==("ARTICLE" OR "REVIEW") AND DT==("ARTICLE" OR "REVIEW") AND LA==("ENGLISH"))

Results: 2710

e. Cochrane Library Search Strategy

#1: ('nuclear magnetic resonance imaging' or 'MRI' or 'Brain MRI' or ('Neuroimaging' or 'Brain Imaging' or 'Cerebral Imaging' or 'Structural Imaging') or ('Diffusion Tensor Imaging' or 'DTI' or 'White Matter Imaging') or ('Susceptibility Weighted Imaging' or 'SWI') or ('Fluid-Attenuated Inversion Recovery Imaging' or 'FLAIR') or ('T2-weighted imaging' or 'T-1-weighted imaging') or ('Positron Emission Tomography' or 'PET Imaging') or ('Computer Assisted Tomography' or 'CT SCAN' or 'CT imaging')).ti,ab.

#2: ('Genetic Predisposition' or 'Genetic Risk' or 'Genetic Variants' or 'Genome-Wide Association Study' or 'GWAS' or ('APOE' or 'Apolipoprotein E' or 'APO E4') or ('CADASIL' or 'NOTCH3') or ('COL4A1' or 'COL4A2') or ('HTRA1' or 'CARASIL') or ('ABCC6' or 'CRPPA' or 'LAMA2') or ('MTHFR' or 'Homocysteine')).ti,ab.

#3: ("Cerebral Small Vessel Diseases" or "Small Vessel Disease" or "Cerebral Microangiopathy" or "White Matter Hyperintensities" or "Lacunar Infarcts" or "Perivascular Spaces" or "Microbleeds" or "Leukoaraiosis").ti,ab.

#4: ("Dementia" or "Cognitive Dysfunction" or "Vascular Dementia" or "Subcortical Ischemic Vascular Dementia" or "Cognitive Impairment" or "Cognitive Decline" or "Vascular Cognitive Impairment”).ti, ab.

Search Query: (#1 OR #2) AND #3 AND #4

Filters: Full systematic reviews

Results: 1

1. **Supplemental Tables**

a. **Supplemental Table S1:** PRISMA-2020 Checklist

Section and Topic	Item #	Checklist item	Location where item is reported
**TITLE**			
Title	1	Identify the report as a systematic review.	1
**ABSTRACT**			
Abstract	2	See the PRISMA 2020 for Abstracts checklist.	3
**INTRODUCTION**			
Rationale	3	Describe the rationale for the review in the context of existing knowledge.	4
Objectives	4	Provide an explicit statement of the objective(s) or question(s) the review addresses.	4
**METHODS**			
Eligibility criteria	5	Specify the inclusion and exclusion criteria for the review and how studies were grouped for the syntheses.	5-6
Information sources	6	Specify all databases, registers, websites, organisations, reference lists and other sources searched or consulted to identify studies. Specify the date when each source was last searched or consulted.	5
Search strategy	7	Present the full search strategies for all databases, registers and websites, including any filters and limits used.	Supplemental Information
Selection process	8	Specify the methods used to decide whether a study met the inclusion criteria of the review, including how many reviewers screened each record and each report retrieved, whether they worked independently, and if applicable, details of automation tools used in the process.	6
Data collection process	9	Specify the methods used to collect data from reports, including how many reviewers collected data from each report, whether they worked independently, any processes for obtaining or confirming data from study investigators, and if applicable, details of automation tools used in the process.	6
Data items	10a	List and define all outcomes for which data were sought. Specify whether all results that were compatible with each outcome domain in each study were sought (e.g. for all measures, time points, analyses), and if not, the methods used to decide which	6

Section and Topic	Item #	Checklist item	Location where item is reported
		results to collect.	
	10b	List and define all other variables for which data were sought (e.g. participant and intervention characteristics, funding sources). Describe any assumptions made about any missing or unclear information.	6
Study risk of bias assessment	11	Specify the methods used to assess risk of bias in the included studies, including details of the tool(s) used, how many reviewers assessed each study and whether they worked independently, and if applicable, details of automation tools used in the process.	6-8
Effect measures	12	Specify for each outcome the effect measure(s) (e.g. risk ratio, mean difference) used in the synthesis or presentation of results.	7
Synthesis methods	13a	Describe the processes used to decide which studies were eligible for each synthesis (e.g. tabulating the study intervention characteristics and comparing against the planned groups for each synthesis (item #5)).	N/A
	13b	Describe any methods required to prepare the data for presentation or synthesis, such as handling of missing summary statistics, or data conversions.	7
	13c	Describe any methods used to tabulate or visually display results of individual studies and syntheses.	7
	13d	Describe any methods used to synthesize results and provide a rationale for the choice(s). If meta-analysis was performed, describe the model(s), method(s) to identify the presence and extent of statistical heterogeneity, and software package(s) used.	7
	13e	Describe any methods used to explore possible causes of heterogeneity among study results (e.g. subgroup analysis, meta-regression).	7
	13f	Describe any sensitivity analyses conducted to assess robustness of the synthesized results.	7
Reporting bias assessment	14	Describe any methods used to assess risk of bias due to missing results in a synthesis (arising from reporting biases).	7

Section and Topic	Item #	Checklist item	Location where item is reported
Certainty assessment	15	Describe any methods used to assess certainty (or confidence) in the body of evidence for an outcome.	6-7
**RESULTS**			
Study selection	16a	Describe the results of the search and selection process, from the number of records identified in the search to the number of studies included in the review, ideally using a flow diagram.	5, Figure 1
	16b	Cite studies that might appear to meet the inclusion criteria, but which were excluded, and explain why they were excluded.	5, Figure 1
Study characteristics	17	Cite each included study and present its characteristics.	6, Tables 1-3
Risk of bias in studies	18	Present assessments of risk of bias for each included study.	6, Supplemental Information
Results of individual studies	19	For all outcomes, present, for each study: (a) summary statistics for each group (where appropriate) and (b) an effect estimate and its precision (e.g. confidence/credible interval), ideally using structured tables or plots.	8-9, Tables 4-7, Supplemental Information
Results of syntheses	20a	For each synthesis, briefly summarise the characteristics and risk of bias among contributing studies.	9-13, Supplemental Information
	20b	Present results of all statistical syntheses conducted. If meta-analysis was done, present for each the summary estimate and its precision (e.g. confidence/credible interval) and measures of statistical heterogeneity. If comparing groups, describe the direction of the effect.	9-13, Tables 4-7, Supplemental Information
	20c	Present results of all investigations of possible causes of heterogeneity among study results.	9-13, Tables 4-7, Supplemental Information
	20d	Present results of all sensitivity analyses conducted to assess the robustness of the synthesized results.	9-13, Tables 4-7, Supplemental Information
Reporting biases	21	Present assessments of risk of bias due to missing results (arising from reporting biases) for each synthesis assessed.	9-12, Supplemental

Section and Topic	Item #	Checklist item	Location where item is reported
			Information
Certainty of evidence	22	Present assessments of certainty (or confidence) in the body of evidence for each outcome assessed.	13, Table 7
**DISCUSSION**			
Discussion	23a	Provide a general interpretation of the results in the context of other evidence.	14-16
	23b	Discuss any limitations of the evidence included in the review.	16
	23c	Discuss any limitations of the review processes used.	17
	23d	Discuss implications of the results for practice, policy, and future research.	16
**OTHER INFORMATION**			
NA	24a	Provide registration information for the review, including register name and registration number, or state that the review was not registered.	NA
	24b	Indicate where the review protocol can be accessed, or state that a protocol was not prepared.	NA
	24c	Describe and explain any amendments to information provided at registration or in the protocol.	NA
Support	25	Describe sources of financial or non-financial support for the review, and the role of the funders or sponsors in the review.	NA
Competing interests	26	Declare any competing interests of review authors.	NA
Availability of data, code and other materials	27	Report which of the following are publicly available and where they can be found: template data collection forms; data extracted from included studies; data used for all analyses; analytic code; any other materials used in the review.	18, Supplementary Information

From: [1] Page MJ, McKenzie JE, Bossuyt PM, Boutron I, Hoffmann TC, Mulrow CD, et al. The PRISMA 2020 statement: an updated guideline for reporting systematic reviews. BMJ 2021;372:n71. doi: 10.1136/bmj.n7

b. Supplemental Table S2: MOOSE Checklist

Item No	Recommendation	Reported on Page No
Reporting of background should include		
1	Problem definition	4
2	Hypothesis statement	NA
3	Description of study outcome(s)	4
4	Type of exposure or intervention used	NA
5	Type of study designs used	4
6	Study population	4
Reporting of search strategy should include		
7	Qualifications of searchers (eg, librarians and investigators)	1
8	Search strategy, including time period included in the synthesis and key words	5, Supplemental Information
9	Effort to include all available studies, including contact with authors	5
10	Databases and registries searched	5, Supplemental Information
11	Search software used, name and version, including special features used (eg, explosion)	5, Supplemental Information
12	Use of hand searching (eg, reference lists of obtained articles)	NA
13	List of citations located and those excluded, including justification	5, Figure 1
14	Method of addressing articles published in languages other than English	5, Figure 1
15	Method of handling abstracts and unpublished studies	5, Figure 1
16	Description of any contact with authors	NA
Reporting of methods should include		
17	Description of relevance or appropriateness of studies assembled for assessing the hypothesis to be tested	5
18	Rationale for the selection and coding of data (eg, sound clinical principles or convenience)	6
19	Documentation of how data were classified and coded (eg, multiple raters, blinding and interrater reliability)	6
20	Assessment of confounding (eg, comparability of cases and controls in studies where appropriate)	6
21	Assessment of study quality, including blinding of quality assessors, stratification or regression on possible predictors of study results	6-7, Supplemental Information
22	Assessment of heterogeneity	7-8, Tables 4-6, Supplemental information
23	Description of statistical methods (eg, complete description of fixed or random effects models, justification of whether the chosen models account for predictors of study results, dose-response models, or cumulative meta-analysis) in sufficient detail to be replicated	7-8
24	Provision of appropriate tables and graphics	Tables 1-7, Figures 2-6

Item No	Recommendation	Reported on Page No
Reporting of results should include		
25	Graphic summarizing individual study estimates and overall estimate	Figures 2-6, Supplemental Information
26	Table giving descriptive information for each study included	Tables 1-3
27	Results of sensitivity testing (eg, subgroup analysis)	9, Supplemental Information
28	Indication of statistical uncertainty of findings	13, Table 7
Reporting of discussion should include		
29	Quantitative assessment of bias (eg, publication bias)	12, Supplemental Information
30	Justification for exclusion (eg, exclusion of non-English language citations)	5, Figure 1
31	Assessment of quality of included studies	6, Supplemental Information
Reporting of conclusions should include		
32	Consideration of alternative explanations for observed results	17
33	Generalization of the conclusions (ie, appropriate for the data presented and within the domain of the literature review)	17
34	Guidelines for future research	17
35	Disclosure of funding source	18

From: Stroup DF, Berlin JA, Morton SC, et al, for the Meta-analysis Of Observational Studies in Epidemiology (MOOSE) Group. Meta-analysis of Observational Studies in Epidemiology. A Proposal for Reporting. JAMA. 2000;283(15):2008-2012. doi: 10.1001/jama.283.15.2008.

c. Supplemental Table S3: Modified Jadad Analysis for Methodological Quality

StudyID	Author	Criteria 1	Criteria 2	Criteria 3	Criteria 4	Criteria 5	Criteria 6	Criteria 7	Criteria 8	Total
1	Brickman et al	0	0	0	0	1	1	0	1	3
2	Chen et al	0	0	0	0	1	1	1	1	4
3	Ding et al	0	0	0	0	1	1	1	1	4
4	Dobrynina et al	0	0	0	0	0	1	1	1	3
5	Fan et al	0	0	0	0	0	1	1	1	3
6	Ferro et al	0	0	0	0	0	1	1	1	3
7	Han et al	0	0	0	0	1	1	1	1	4
8	Hilal et al	0	0	0	0	1	1	1	1	4
9	Hilal et al	0	0	0	0	1	1	1	1	4
10	Hong et al	0	0	0	0	0	1	1	1	3
11	Jacob et al	0	0	0	0	1	1	1	1	4
12	Ke et al	0	0	0	0	0	1	1	1	3
13	Kim et al	0	0	0	0	0	1	1	1	3
14	Lee et al	0	0	0	0	1	1	1	1	4
15	Li et al	0	0	0	0	0	1	1	1	3
16	Li et al	0	0	0	0	0	1	1	1	3
17	Liao et al	0	0	0	0	0	1	1	1	3
18	Liu et al	0	0	0	0	0	1	1	1	3
19	Nicoll et al	0	0	0	0	0	0	1	1	2
20	Paradela et al	0	0	0	0	0	1	1	1	3
21	Paradise et al	0	0	0	0	1	1	1	1	4
22	Rennie et al	0	0	0	0	0	1	1	1	3

23	Romero et al	0	0	0	0	1	1	1	1	4
24	Shaikh et al	0	0	0	0	0	1	1	1	3
25	Song et al	0	0	0	0	0	1	1	1	3
26	Sun et al	0	0	0	0	0	1	1	1	3
27	Tang et al	0	0	0	0	0	1	1	1	3
28	Uetani et al	0	0	0	0	0	1	1	1	3
29	Wang et al	0	0	0	0	0	1	1	1	3
30	Wang et al	0	0	0	0	0	1	1	1	3
31	Wang et al	0	0	0	0	0	1	1	1	3
32	Wei et al	0	0	0	0	0	1	1	1	3
33	Wrigley et al	0	0	0	0	0	1	0	1	2
34	Xing et al	0	0	0	0	0	1	1	1	3
35	Xu et al	0	0	0	0	0	1	1	1	3
36	Yu et al	0	0	0	0	0	1	1	1	3
37	Zhu et al	0	0	0	0	0	1	1	1	3
38	Zhu et al	0	0	0	0	0	1	1	1	3

Criteria 1: Was the study randomised? (0 = not described or no, 1 = yes)
Criteria 2: Was the method of randomisation appropriate (0 = not described or no, 1 = yes)
Criteria 3: Was the study described as being blinded? (0 = not described or no, 0.5 = single blinded 1 = double-blinded)
Criteria 4: Was the method of blinding appropriate (0 = not described or no, 1 = yes)
Criteria 5: Was there a description of withdrawals and dropouts? (0 = not described or no, 1 = yes)
Criteria 6: Was there a clear description of the inclusion/exclusion criteria? (0 = not described or no, 1 = yes)
Criteria 7: Was the method used to assess adverse events described? (0 = not described or no, 1 = yes)
Criteria 8: Was the method of statistical analysis described? (0 = not described or no, 1 = yes)

d. Supplemental Table S4: Funding Bias Scores for Studies

StudyID	Author	Publication Bias	Funding
1	Brickman et al	2	Supported by the National Institues of Health, regional government of Languedoc-Roussillon, the Agence Nationale de la Recherche, an unconditional grant from Novartis and France Alzheimer.
2	Chen et al	0	*Supported by the Medical Scientific Research Foundation of Guangdong Province, China.*
3	Ding et al	1	Supported by the National Institues of Health, Intramural Research Program of the National Institute on Aging, Icelandic Heart Association, and Icelandic Parliament
4	Dobrynina et al	0	Supported by the Russian Science Foundation.
5	Fan et al	1	Supported by the National Health Commission and the Beijing Natural Science Foundation.
6	Ferro et al	1	Supported by the Dutch Heart Association, ZonMw, the Netherlands Organization for Health Research and Development, and the Netherlands Organization for Scientific Research.
7	Han et al	1	Supported by the Shanghai Jiao Tong University, the National Natural Science Foundation of China, the Shanghai Science and Technology Committee Project, the Shanghai Municipal Health Commission and the Shanghai Shenkang Hospital Development Center.
8	Hilal et al	0	An author received support from the Singapore Ministry of Health's National Medical Research Council.
9	Hilal et al	1	Supported by the National Medical Research Council (NMRC), Singapore and Bight Focus Foundation, the Ministry of Health, Singapore, and Center for Sleep and Cognition funded by Yong Loo Lin School of Medicine, National University of Singapore.
10	Hong et al	0	Supported by a grant of the Korea Healthcare Technology Research and Development Project, Ministry for Health, Welfare & Family Affairs, Republic of Korea.
11	Jacob et al	2	An author is local principal investigator for the EVOKE study sponsored by Novo Nordisk, has served as a consultant for Roche, a speaker for Bayer and Sanofi, and as an adjudication board member for Hovid Berhad. Two authors received support from the Dutch Brain Foundation and the Netherlands Organization for Scientific Research. An author is supported by the China Scholarship Council.

12	Ke et al	1	Supported by Brain Science and Brain-Like Research, the National Natural Science Foundation of China, the National Natural Science Foundation of China, the Key Research and Development Program of Jiangsu Province of China, the National Key Research and Development Program of China, the Jiangsu Province Key Medical Discipline .
13	Kim et al	1	Supported by grants from the Korean Healthcare Technology R&D Project, the Ministry for Health, Welfare & Family Affairs, the Republic of Korea, by the Korean Science and Engineering Foundation (KOSEF) NRL program grant funded by the Korean government, by Samsung Medical Center Clinical Research Development Program grants and by the Converging Research Center Program through the Ministry of Education, Science and Technology. Two authors received support from the Ministry of Health and Welfare, Korea.
14	Lee et al	1	Supported by National Research Foundation of Korea grant funded by the Korea government, the Original Technology Research Program for Brain Science, the Korea Ministry of Environment as the Environmental Health Action Program and the Brain Research Program.
15	Li et al	0	Supported by the National Natural Science Foundation of China and Beijing Municipal Administration of Hospitals' Youth Program.
16	Li et al	1	Supported by Erasmus Medical Center and Erasmus University, the Netherlands Organization for Health Research and Development, the Research Institute for Diseases in the Elderly, the Ministry of Education, Culture and Science, the Ministry for Health, Welfare and Sports, the European Commission, and the Municipality of Rotterdam.
17	Liao et al	0	Supported by grants from the Guangzhou Science and Technology Program Key Project and the National Natural Science Foundation of China.
18	Liu et al	0	None reported
19	Nicoll et al	0	Supported by the Medical Research Council (MRC).
20	Paradela et al	1	Supported by grants from the University of Sao Paulo. An author received support from the Alzheimer's Association and National Council for Scientific and Technological Development.
21	Paradise et al	1	Supported by the National Health and Medical Research Council (NHMRC) of Australia. An author received support from the Australian National University. Two authors were supported by NHMRC Program Grant.
22	Rennie et al	2	Authors have served as consultants for Geras Solutions, Optoceutics, Biogen and Bioarctic, H. Lundbeck, Eisai and Evonik. An author have served as a principal investigator in clinical trials sponsored by Biogen, Roche, Axovant and Eisai. Authors have received research support and/or honoraria from AstraZeneca, H. Lundbeck, Novartis Pharmaceuticals, GE Health and Esteve.

			An author has an advisory board membership for BioArctic, Biogen, Combinostics and Nestlé and has received speaking and lecture fees for Biogen, Nestlé, Nutricia and Roche.<br><br>The research was supported by numerous Swedish foundations, councils, and regional health authorities, including the Swedish Research Council, Karolinska Institutet agreements, various medical and neuroscience foundations, dementia and Alzheimer's organizations, private foundations, and regional bodies in Sweden and Norway.
23	Romero et al	1	Supported by the Framingham Heart Study's National Heart, Lung, and Blood Institute, the National Institute of Neurological Disorders and Stroke, the National Institute on Aging, NIH grant and NHLBI grants.
24	Shaikh et al	1	Supported by the Canadian Consortium on Neurodegeneration of Aging, the Canadian Institutes of Health Research, Brain Canada, Canadian Stroke Network, Heart and Stroke Foundation of Alberta and the Alzheimer Society of Canada. An author is supported by the Canada Research Chairs program.
25	Song et al	0	Supported by The Central Government Guiding Funds for Local Science and Technology Development in Hubei Province of China.
26	Sun et al	1	An author is the director of BrainNow Research Institute.
27	Tang et al	0	Supported by the National Natural Science Foundation of China and the Scientific Research Plan Project of Shanghai Science and Technology Committee. An author was supported by the Huaxiu Talents Program of Huashan Hospital.
28	Uetani et al	0	None reported
29	Wang et al	0	Supported by the National Natural Science Foundation of China and the Science Technology Department of Zhejiang Province.
30	Wang et al	1	Supported by the National Key R&D Program of China, the National Nature Science Foundation of China, the Academic Promotion Program of Shandong First Medical University, the Shandong Provincial Key Research and Development Program and the Taishan Scholar Program of Shandong Province. This work was supported by additional grants from the STI2030-Major Project, the Nature Science Foundation of Shandong Province, and Integrated Traditional Chinese and Western Medicine Program in Shandong Province. An author received grants

			from the Swedish Research Council, the Swedish Foundation for International Cooperation in Research and Higher Education, and the Karolinska Institutet, Stockholm, Sweden.
31	Wang et al	0	Supported by the by the National Natural Science Foundation of China, Beijing Outstanding Young Scientist Program, Capital's Funds for Health Improvement and Research, National Key R&D Program of China, Youth Beijing Scholar Program, Beijing Laboratory of Oral HealthBeijing Talent Project - Class A: Innovation and Development , National Ten-Thousand Talent Plan - Leadership of Scientific and Technological Innovation and National Key R&D Program of China.
32	Wei et al	0	Supported by the Beijing Municipal Administration of Hospitals Clinical Medicine Development of Special Funding Support, the National Natural Science Foundation of, the National Key Research and Development Plan, the National Key Technology Research and Development Program of the Ministry of Science and Technology of The People's Republic of China, Beijing Institute For Brain Disorders, the Beijing Municipal Science & Technology Commission, and Beijing Municipal Administration of Hospitals' Youth Programme.
33	Wrigley et al	0	None reported
34	Xing et al	0	Supported by National Natural Science Foundation of China and National Key R&D Program of China.
35	Xu et al	0	None reported
36	Yu et al	0	Supported by the Ministry of Science and Technology of Taiwan and Cardinal Tien Hospital.
37	Zhu et al	1	Supported by the Anhui Provincial Higher Education Scientific Research Project and the Natural Science Foundation of Bengbu Medical College.
38	Zhu et al	2	Supported by the National Natural Science Foundation of China, the Natural Science Foundation of Hubei Province, Beijing Natural Science Funds for Distinguished Young Scholars and the Flagship Program of Tongji Hospital, China. Additional support received from the Alzheimer's Disease Neuroimaging Initiative (ADNI). The ADNI was funded by the National Institute on Aging, the National Institute of Biomedical Imaging and Bioengineering, and generous contributions from AbbVie, Alzheimer's Association; Alzheimer's Drug Discovery Foundation; Araclon Biotech; BioClinica, Inc.; Biogen; Bristol-Myers Squibb Company; CereSpir, Inc.; Cogstate; Eisai Inc.; Elan Pharmaceuticals, Inc.; Eli Lilly and Company; EuroImmun; F. Hoffmann-La Roche Ltd, and its

			affiliated company Genentech, Inc.; Fujirebio; GE Healthcare; IXICO Ltd.; Janssen Alzheimer Immunotherapy Research & Development, LLC.; Johnson & Johnson Pharmaceutical Research & Development LLC.; Lumosity; Lundbeck; Merck & Co., Inc.; Meso Scale Diagnostics, LLC.; NeuroRx Research; Neurotrack Technologies; Novartis Pharmaceuticals Corporation; Pfizer Inc.; Piramal Imaging; Servier; Takeda Pharmaceutical Company; and Transition Therapeutics.<br><br>The Canadian Institutes of Health Research provided funds to support ADNI clinical sites in Canada. Private sector contributions were facilitated by the Foundation for the National Institutes of Health. The grantee organization was the Northern California Institute for Research and Education, and the study was coordinated by the Alzheimer's Therapeutic Research Institute at the University of Southern California. ADNI data were disseminated by the Laboratory for Neuro Imaging at the University of Southern California.
39	Zonneveld et al	2	Supported by Alzheimer Nederland and Stichting VUmc fonds. The clinical database structure was developed with funding from Stichting Dioraphte.<br><br>An author received consultant/and or lecture fees for Biogen Idec, Bayer Healthcare, Roche, and Janssen-Cilag. An author serves on the advisory board of Boehringer Ingelheim, Envivo and has been a speaker for Janssen and Novartis. An author has a senior fellowship at the Alzheimer Center VUmc partly supported by Vereniging AEGON and receives research support from the Brain Foundation of the Netherlands and Alzheimer Nederland. An author receives grant support from GE Healthcare, Danone Research, and Merck and speaker's fees from Lilly, GE Healthcare, Lundbeck, Danone, and Janssen AI-Pfizer. An author received grant funds from the Dutch MS Society and EU-FP7, and consultant fees from Bayer Schering Pharma, Sanofi-Aventis, Biogen Idec, TEVA, Merck-Serono, Novartis, Roche, Synthon BV, and Janssen Research, and presentation fees for the Serono Symposium Foundation.

0 = low risk of bias/funded by government organisation
1 = funded by organisation outside of industry
2 = funded by organisation in industry
3 = high risk of bias

e. Supplemental Table S5: Summary Data and Performance Estimates for Meta-Analysis on the Diagnostic Accuracy of CSVD Neuroimaging Markers and APOE ε4 Allele Carrier Status and Cognitive Outcomes

	**Number of studies**	**Deviance**	**ICC_SEN [95% CI]**	**ICC_SPE [95% CI]**	**Heterogeneity (χ^2, df, p-value)**	**Inconsistency (I^2 [95% CI])**	**AUROC [95% CI]**	**Sensitivity [95% CI]**	**Specificity [95% CI]**	**Positive Likelihood Ratio [95% CI]**	**Negative Likelihood Ratio [95% CI]**	**Diagnostic Odds Ratio [95% CI]**
CMB												
MCI	8	111.3	0.05 [0.00; 0.11]	0.04 [0.00; 0.09]	26.890, df = 2.00, p < 0.001	93 [86; 99]	0.63 [0.58-0.67]	0.33 [0.26; 0.40]	0.79 [0.74; 0.84]	1.6 [1.3; 1.9]	0.85 [0.78; 0.92]	2 [1; 3]
CI	5	80.7	0.12 [0.00; 0.27]	0.36 [0.01; 0.70]	84.796, df = 2.00, p < 0.001	98, [96; 99]	0.52 [0.48-0.56]	0.38 [0.25; 0.53]	0.76 [0.48; 0.92]	1.6 [0.8; 3.1]	0.81 [0.68; 0.96]	2 [1; 4]
ACD	9	140.4	0.10 [0.00; 0.20]	0.06 [0.00; 0.12]	53.272, df = 2.00, p < 0.001	96 [93; 99]	0.66 [0.61-0.70]	0.33 [0.24; 0.43]	0.80 [0.74; 0.85]	1.7 [1.3; 2.0]	0.84 [0.75; 0.93]	2 [1; 3]
VaD	5	68.6	0.29 [0.00; 0.60]	0.04 [0.00; 0.10]	26.403, df = 2.00, p < 0.001	92 [86; 99]	0.81 [0.78-0.85]	0.50 [0.25; 0.75]	0.83 [0.77; 0.87]	2.9 [2.0; 4.2]	0.61 [0.37; 1.00]	5 [2; 11]
AD	7	107.1	0.14 [0.00; 0.31]	0.05 [0.00-0.11]	39.045, df = 2.00, p < 0.001	95 [91; 99]	0.69 [0.65-0.73]	0.22 [0.13; 0.35]	0.84 [0.78; 0.88]	1.3 [1.0; 1.9]	0.93 [0.84; 1.04]	1 [1; 2]
WMH												
MCI	4	67.2	0.33 [0.00; 0.65]	0.28 [0.00; 0.57]	240.557, df = 2.00, p < 0.001	99 [99; 100]	0.65 [0.61; 0.69]	0.57 [0.27, 0.82]	0.66 [0.38, 0.86]	1.7 [1.3; 2.2]	0.66 [0.46; 0.95]	3 [2; 4]
Lacunes												
MCI	4	60.9	0.04 [0.00; 0.11]	0.36 [0.00; 0.77]	54.276, df = 2.00, p < 0.001	96 [94; 99]	0.44 [0.40; 0.49]	0.32 [0.23, 0.41]	0.89 [0.65, 0.97]	2.9 [0.9; 8.7]	0.77 [0.69; 0.86]	4 [1; 12]
CI	5	73.4	0.12 [0.00; 0.26]	0.29 [0.00; 0.57]	130.376, df = 2.00, p < 0.001	98 [98; 99]	0.60 [0.55; 0.64]	0.40 [0.27, 0.56]	0.81 [0.60, 0.93]	2.2 [1.2; 3.8]	0.73 [0.67; 0.81]	3 [2; 5]
ACD	4	59.5	0.03 [0.00; 0.09]	0.44 [0.00; 0.94]	37.655, df = 2.00, p < 0.001	95 [90; 99]	0.45 [0.40; 0.49]	0.33 [0.25, 0.43]	0.90 [0.61, 0.98]	3.4 [0.8; 14.2]	0.74 [0.65; 0.83]	5 [1; 21]
APOE ε4 allele												
CI	6	79.6	0.02 [0.00; 0.06]	0.00 [0.00; 0.01]	5.216, df = 2.00, p = 0.037	62 [14; 100]	0.69 [0.65; 0.73]	0.35 [0.29, 0.41]	0.76 [0.73, 0.79]	1.5 [1.2; 1.7]	0.86 [0.79; 0.93]	2 [1; 3]

f. Supplemental Table S6: Outputs from Egger's Regression Test

	Std_Eff	Coefficient [95% CI]	Std. Err.	t	P>|t|	Test of H0: no small-study effects
CMBs	Slope	0.065 [-0.37; 0.50]	0.21	0.3	0.76	0.013
	Bias	1.73 [0.40; 3.06]	0.65	2.64	0.01	
WMHs	Slope	0.71 [0.02; 1.40]	0.30	2.37	0.05	0.23
	Bias	1.97 [-1.52; 5.45]	1.51	1.3	0.23	
Lacunes	Slope	0.01 [-0.53; 0.54]	0.24	0.03	0.97	0.011
	Bias	2.49 [0.69; 4.28]	0.82	3.05	0.01	
APOE ε4 allele	Slope	0.061 [-0.84; 2.06]	0.52	1.17	0.31	0.97
	Bias	-0.075 [-5.52; 5.37]	1.96	-0.04	0.97	

g. Supplemental Table S7: Outputs from Deek's Funnel-Plot Asymmetry Test

	yb	Coefficient [95% CI]	Std. Err.	t	P>|t|
CMBs	Bias	9.70 [0.32; 19.07]	4.60	2.11	0.04
	Intercept	0.00 [-0.63; 0.64]	0.31	0.01	1.00
WMHs	Bias	12.15 [-3.36; 27.67]	6.73	1.81	0.11
	Intercept	0.60 [-0.02; 1.22]	0.27	2.23	0.06
Lacunes	Bias	20.89 [0.10; 41.68]	9.45	2.21	0.05
	Intercept	-0.20 [-1.50; 1.09]	0.59	-0.35	0.74
APOE ε4 allele	Bias	1.51 [-23.74; 26.76]	9.09	0.17	0.88
	Intercept	0.49 [-1.04; 2.01]	0.55	0.89	0.43

2. Supplemental Figures

a. **Supplemental Figure S1:** Summary Receiver Operating Characteristic (SROC) Curves for Imaging Markers of CSVD and APOE ε4 Allele Carrier Status (1)

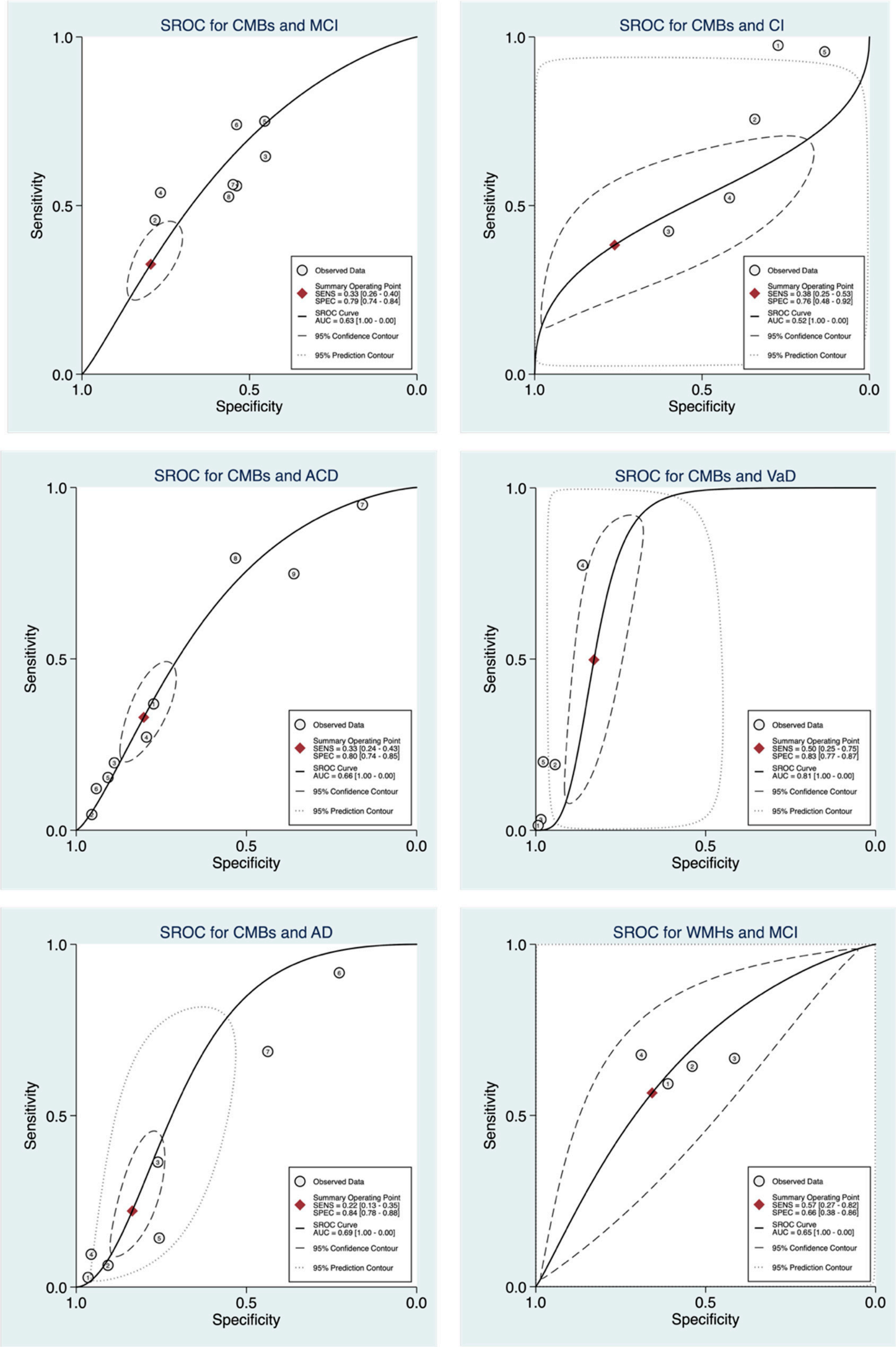

b. Supplemental Figure S2: Summary Receiver Operating Characteristic (SROC) Curves for Imaging Markers of CSVD and APOE ε4 Allele Carrier Status (2)

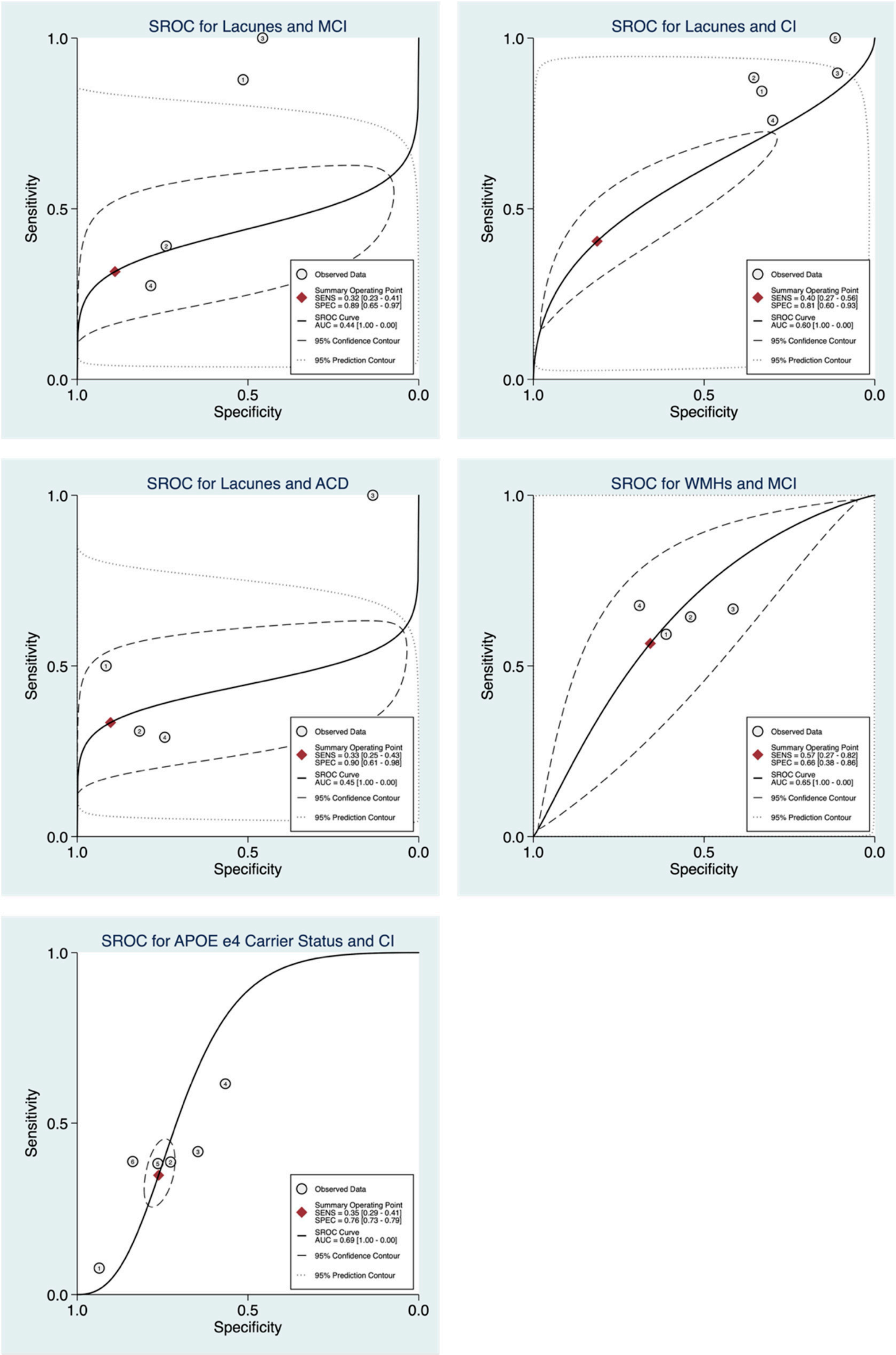

c. Supplemental Figure S3: Sensitivity Analysis for Meta-analysis on the Association between CSVD Neuroimaging Markers and APOE ε4 Allele Carrier Status and Cognitive Outcomes

a) Association between CMBs and Cognitive Outcomes

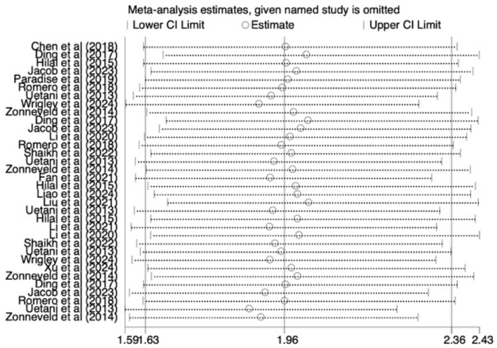

b) Association between WMHs and Cognitive Outcomes

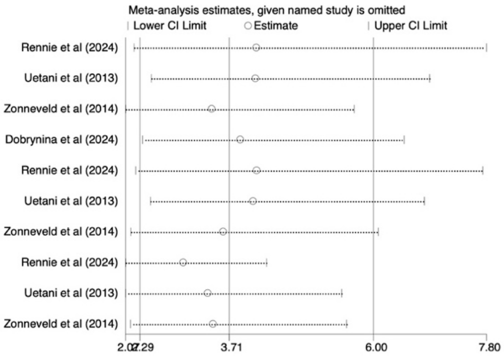

c) Association between Lacunes and Cognitive Outcomes

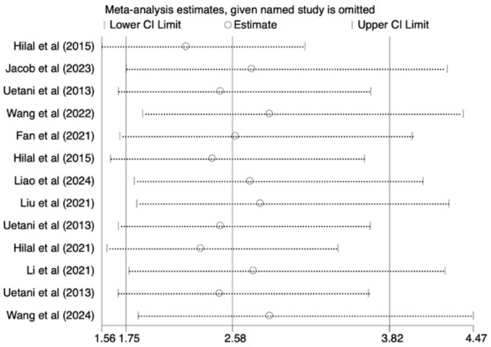

d) Association between APOE ε4 Allele Carrier Status and Cognitive Outcomes

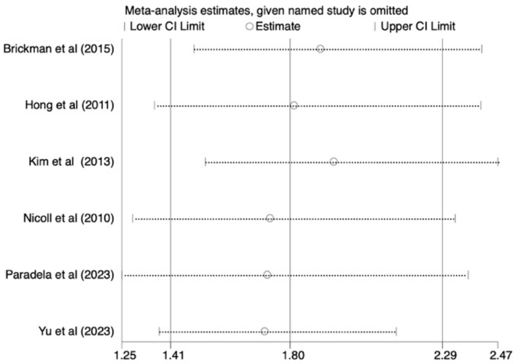

d. **Supplemental Figure S4:** Graphs of Egger's Regression Test

a) Association between CMBs and Cognitive Outcomes

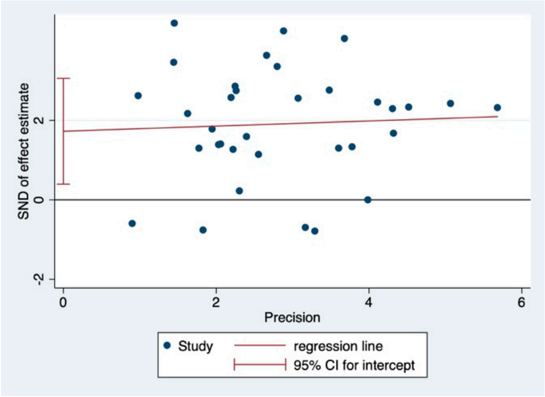

b) Association between WMHs and Cognitive Outcomes

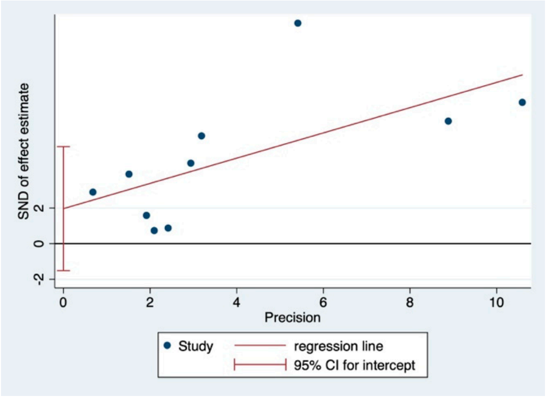

c) Association between Lacunes and Cognitive Outcomes

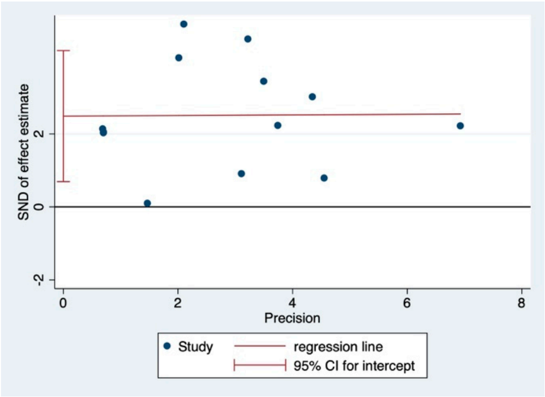

d) Association between APOE ε4 Allele Carrier Status and Cognitive Outcomes

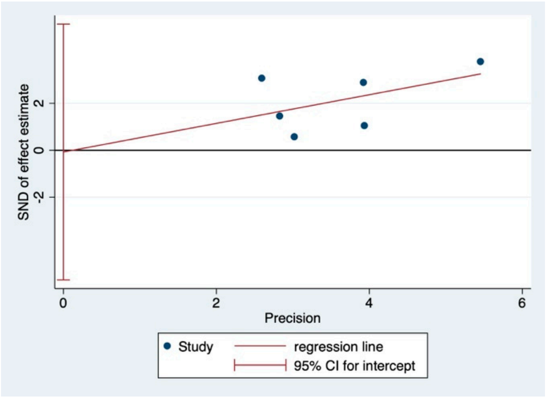

e. **Supplemental Figure S5:** Graphs of Funnel Plots

a) Association between CMBs and Cognitive Outcomes

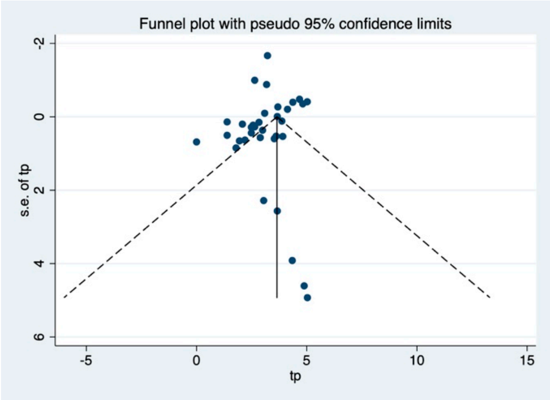

b) Association between WMHs and Cognitive Outcomes

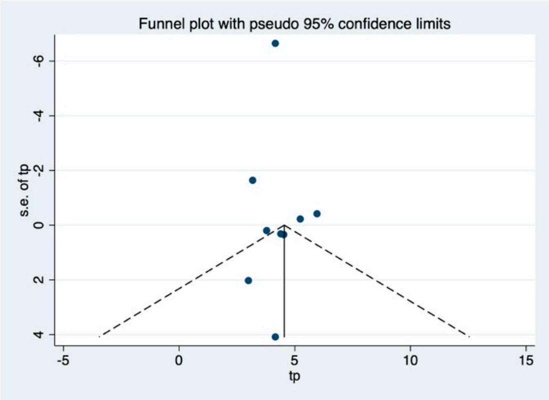

c) Association between Lacunes and Cognitive Outcomes

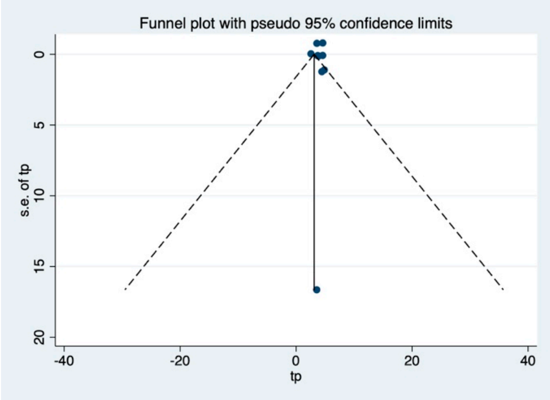

d) Association between APOE ε4 Allele Carrier Status and Cognitive Outcomes

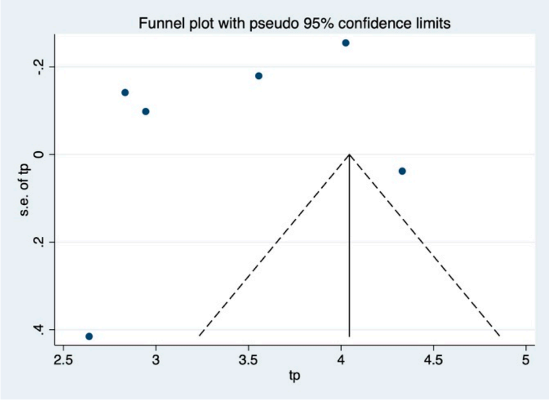

f. Supplemental Figure S6: Graphs of Deek's Funnel-Plot Asymmetry Test

a) CMBs and Cognitive Outcomes

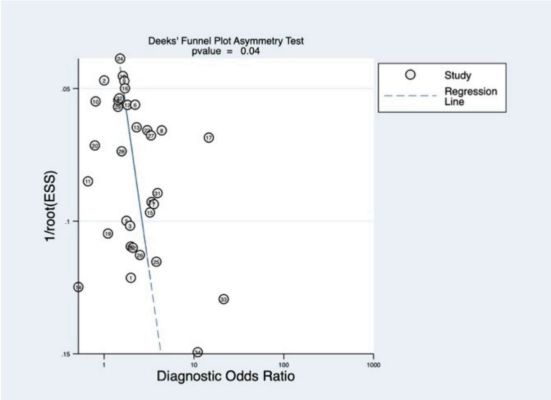

b) WMHs and Cognitive Outcomes

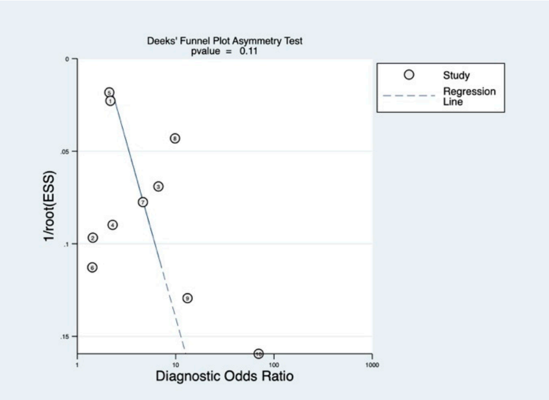

c) Lacunes and Cognitive Outcomes

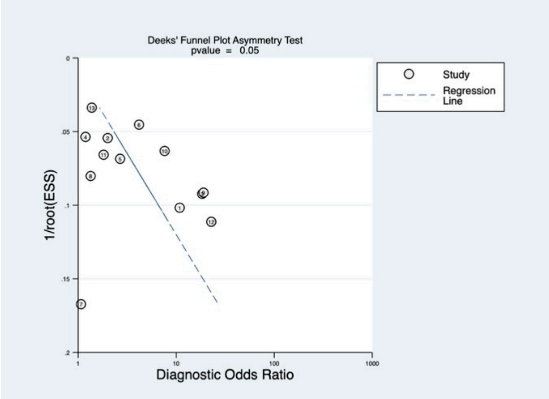

d) APOE ε4 Allele Carrier Status and Cognitive Outcomes

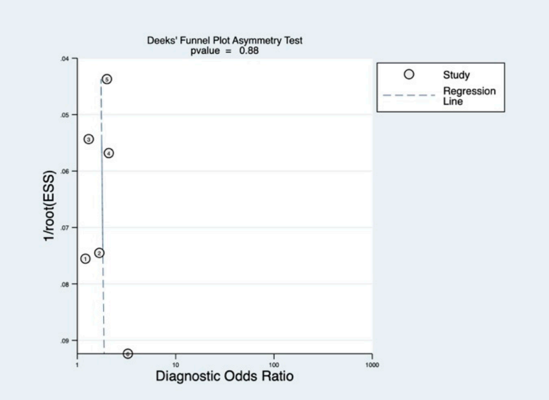

g. **Supplemental Figure S7:** Graphs of Fagan's Normogram

a) CMBs and Cognitive Outcomes

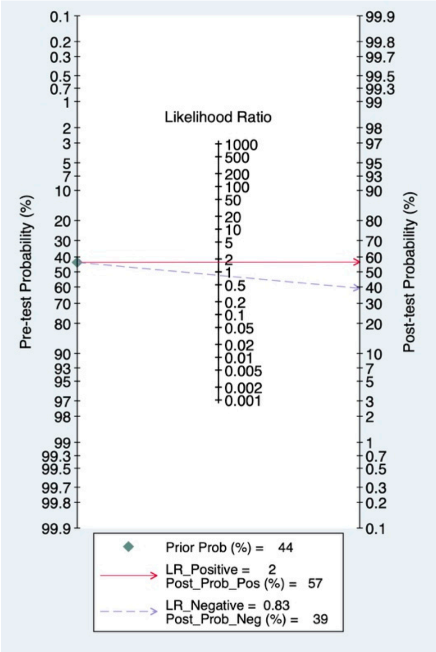

b) WMHs and Cognitive Outcomes

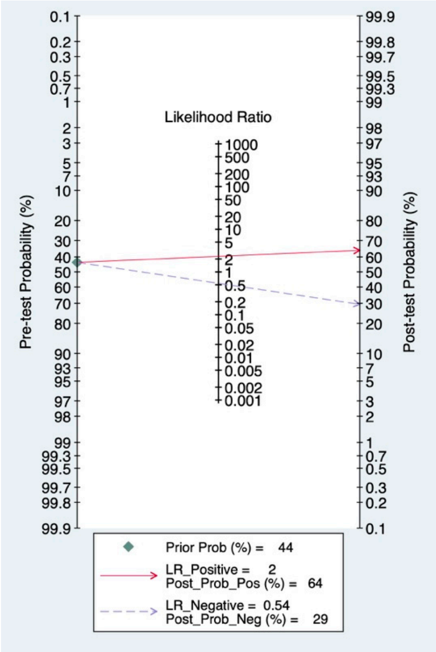

c) Lacunes and Cognitive Outcomes

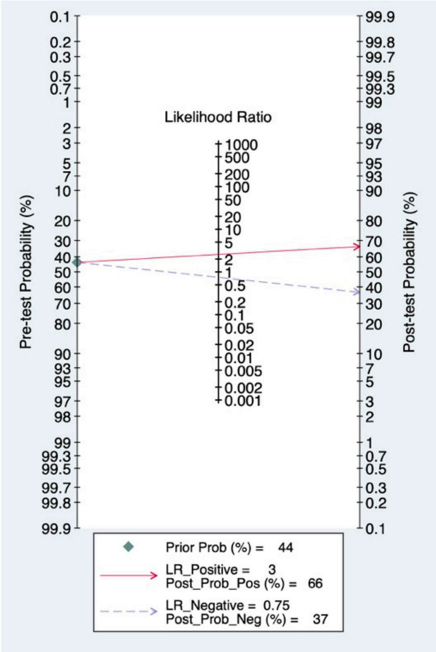

d) APOE ε4 Allele Carrier Status and Cognitive Outcomes

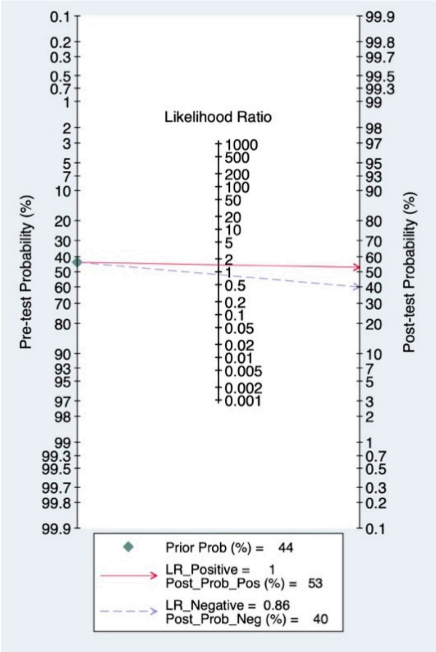

Supplement: Supplementary file 1 [file diagnostics-15-02585-s001.zip › diagnostics-3884282-supplementary.pdf]
